# Do trophic strategies shape biogeography and environmental niches? Marine dinoflagellates as a case study

**DOI:** 10.1093/ismeco/ycaf153

**Published:** 2025-09-16

**Authors:** Gaspard Rihm, Fabio Benedetti, Lucie Bittner

**Affiliations:** Institut de Systématique, Evolution, Biodiversité (ISYEB), Muséum National d'Histoire Naturelle, CNRS, Sorbonne Université, EPHE-PSL, Université des Antilles, Paris 75005, France; Environmental Physics (UP) Group, Institute of Biogeochemistry and Pollutant Dynamics, ETH Zürich, 8092 Zürich, Switzerland; Plant Ecology Group, Institute of Plant Sciences, University of Bern, 3013 Bern, Switzerland; Institut de Systématique, Evolution, Biodiversité (ISYEB), Muséum National d'Histoire Naturelle, CNRS, Sorbonne Université, EPHE-PSL, Université des Antilles, Paris 75005, France; Institut Universitaire de France, Paris 75005, France

**Keywords:** dinoflagellates, trophic strategy, SDM, biogeography, mixotrophy, phagotrophy

## Abstract

Marine unicellular eukaryotes (protists) exhibit a wide spectrum of trophic strategies ranging from specialists (strict phototrophy or strict phagotrophy) to generalist (mixotrophy). Generalist strategies enable flexibility in nutrient sources, which impacts biogeochemical cycles, energy fluxes in planktonic food webs as well as species biogeography. Dinoflagellates exhibit specialist and generalist trophic strategies, making them a key group for studying the ecological success of trophic traits from a biogeographical perspective. Yet, our understanding of what drives their biogeography remains limited although they are a major component of planktonic communities. Here, we combine one of the largest environmental genomics databases with state-of-the-art species distribution modelling to test whether trophic dinoflagellate specialists exhibit distinct spatial distributions and abiotic drivers compared to generalists. Based on field observations alone, we find that dinoflagellate species show similar abundance and evenness patterns, regardless of their trophic strategies. However, our models reveal differences in environmental niches at the trait level: mixotrophy is favoured in tropical oligotrophic regions whereas strict phagotrophy is favoured in the productive high-latitudes. At the species level, mixotrophs show similar responses across gradients of nutrient availability, whereas species responses to abiotic gradients are more divergent within strict phagotrophs. The latter pattern is consistent with a trait scenario of multiple evolutionary convergences. We show that trophic classification effectively explains the distribution patterns and environmental responses of generalists but is less effective in capturing the diverse responses of specialists that could result from other factors (evolutionary history, biotic interactions, cell size).

## Introduction

In the oceans, planktonic microorganisms sustain numerous ecological functions and ecosystem services, including oxygen production, carbon fixation, and underpin the basis of food webs. They influence biogeochemical cycles through their interactions and metabolisms, while environmental conditions, in turn, shape their physiology and behavior [1, 2]. During the last decade, high-throughput sequencing highlighted the extraordinary biodiversity of unicellular eukaryotes (protists) in terms of species richness [3–6], functions, and ecological interactions [7–9]. Protist sequences assigned to “dinoflagellates” are prominent in the pico- to meso-plankton size fractions [5, 10–12]. Dinoflagellates correspond to organisms belonging to the class Dinophyceae that comprises more than 2300 morphological species, 82% of which are found in marine planktonic ecosystems [13]. Several species play major ecological roles including the formation of toxic algal blooms (e.g. *Alexandrium*) and symbiosis with coral reefs (e.g. *Symbiodinium*). They can dominate coastal and open oceans communities (e.g. *Prorocentrum* cf. *balticum*, *Gonyaulax* sp., *Noctiluca scintillans*, [8, 14]) despite their relatively slow growth rates and limited efficiency in nutrient uptake [15, 16]. This success was hypothesized as linked to their diverse trophic strategies and physiological flexibility [17–19].

Many dinoflagellates are indeed mixotrophs, i.e. performing photo-autotrophy and phago-heterotrophy within the same cell. This generalist trait allows trophic flexibility as a response to environmental perturbations [20]. Mixotrophy is facultative or obligatory, and is either acquired constitutively (Constitutive mixotrophs, phototrophic lineages with vertically inherited plastids, capable of phagotrophy, later called CM) or non-constitutively (Non-constitutive mixotrophs, later called NCM, phago-heterotrophic lineages acquiring plastids from other lineages by specific [pSNCM, i.e. plastidic specialists] or non-specific [GNCM, i.e. generalists] kleptoplasty, or via endosymbiosis [eSNCM, i.e. endosymbiotic specialists]) [21, 22]. Additionally to these trophic-generalist strategies, dinoflagellates also involve trophic-specialist species: i.e. strict phago-heterotophs and strict phototrophs (Fig. S1). Trophic annotation relies on traits observations (in-situ or in-vitro) or ingestion rates measurements [23, 24]. Mixotrophy is a flexible trait and depends on the environmental context [25], only a small part of phago-mixotrophic species are actually successfully cultured [23].

Over the past five years, the concept of trophic flexibility became central to marine biogeochemical cycles and biogeographical studies, but its integration in biogeochemical models remains challenging [14, 24, 26–28]. Experimental methods showed notably that trophic realisation within a lineage depends on intrinsic (e.g. evolutionary history of trophic mode acquisition, its degree of dependence to mixotrophy, prey-process duration), and extrinsic biotic factors (e.g. biotic: presence of preys, and abiotic: nutrients availability, temperature, light) [29, 30]. Meanwhile, theoretical models predict that mixotrophy is advantageous in oligotrophic environments whereas trophic specialists are better suited to nutrient-rich regions of the surface ocean due to their higher growth efficiency in productive environments [31–33]. The development of open access repositories of geolocated biodiversity data (e.g. OBIS) and large-scale sampling cruises from planktonic communities (e.g. Tara Oceans, Malaspina, Ocean Sampling Day) offers the opportunity of exploring (semi-)quantitatively, at quasi global scale, the impact of extrinsic factors on species biogeography and their link to trophic strategies. Studies focusing on mixotrophic protists highlighted that CM are present in environmental conditions ranging from shallow eutrophic waters to oligotrophic regions, and that NCM distributions were narrower, and depended on the specificity and mode of plastids acquisition [34, 35]. These studies, however, ignored the potential strong divergence between species sharing the same trophic strategies. Relying on both growth-rates measurements and in-situ metabarcoding data, Edwards et al., [25] highlighted niche differences for a spectrum of mixotrophic nanoplanktonic species along resource gradients, suggesting the need to take into account such diversity.

Species distribution models (SDMs) are data-driven methods that allow to fit simple to complex relationships between biological response variables and abiotic covariates [36]. These models are suitable to investigate the diversity of species responses and their traits to environmental gradients. Previous studies of the entire planktonic community predicted a strong latitudinal gradient in global dinoflagellates diversity, with higher values in eastern boundary upwelling systems and lower ones in tropical oligotrophic gyres [37, 38]. However, these models considered dinoflagellates as a single group, ignoring their heterogeneous trophic strategies and their potential influence on species biogeography.

In this study, we assess whether the biogeography and ecological success (here defined through indices of ubiquity and dominance based on field observations of metabarcoding reads) of dinoflagellates in marine planktonic communities is driven by their trophic strategy. Then, based on the same metabarcoding data, we train SDMs to explore the variability of geographical ranges across dinoflagellate trophic strategies in the global surface ocean. Our study aims to uncover patterns of dinoflagellates distribution based on their trophic strategies, highlight oceanic regions where different trophic strategies coexist or occur independently and test whether trophic traits emerge as significant determinants of biogeographical patterns. We test the following hypotheses: generalist trophic strategies should be more ubiquitous, and especially present in nutrient-depleted regions such as the oligotrophic low-latitude oceanic biomes whereas trophic specialists should be more geographically restricted, and favoured in nutrient-rich regions such as the high-latitude productive oceanic biomes.

## Materials and methods

### Data collection and trophic annotation

The metaPR2 metabarcoding database was used (https://shiny.metapr2.org/metapr2/; [39]) to select Amplicon Sequence Variants (ASVs) obtained from environmental DNA collected in marine pelagic ecosystems. This database gathers more than 41 datasets of processed 18S rRNA metabarcodes associated with a taxonomic annotation performed with the PR^2^ reference sequence database [40]. ASVs showing a minimum number of 100 reads (according to the minimum default parameters) and corresponding to the V4 region were kept without distinction of size class. In total, 3242 ASVs from 251 different lineages (i.e. distinct taxonomic affiliation) were identified as belonging to the Dinophyceae class based on the taxonomy assignments of the PR2 database. The 3242 ASVs retained came from 2776 field DNA samples corresponding to 895 unique sampling sites. The samples’ metadata (e.g. latitude and longitude, size range of the sampled fraction, sampling date, depth) were also retrieved from metaPR2. In order to focus the study on areas with sufficient light availability, only samples from the euphotic zone and located above the climatological mixed layer depth (MLD) were kept. Six lineages out of the 251 were removed because they were not taxonomically assigned at least at the genus level. The resulting matrix describes the relative abundance of 245 lineages (later called “species” to facilitate further results interpretation) across 895 sites. Abundance corresponds to the number of reads assigned to the lineage. Seven classes were retained for trophic annotation based on the traits databases [23, 41].

### Environmental data

For each sampling site, monthly climatological layers of environmental variables were collected. These variables are relevant to model the biogeography of marine protists as they shape the spatial distribution and control the physiology of these microorganisms [37, 42, 43]. The following variables were mainly taken from Knecht et al., [44] and mostly sourced from the World Ocean Atlas 2018: Sea Surface Temperature (SST; °C), Sea Surface Salinity (SSS; unitless), Mixed Layer Depth (MLD; m), Photosynthetically Active Radiation (PAR; μmol m-2.s-1), Dissolved Oxygen Concentration (O2; μmol kg-1), Surface Particulate Inorganic Carbon Concentration (PIC; mol.m-3), Surface Chlorophyll-a Concentration (Chla; mg.m-3), Surface Nitrates Concentration (NO3; μmol.kg-1), Surface Phosphates Concentration (PO4; μmol kg-1) and Surface Silicates Concentration (Si; μmol kg-1). From the nutrient concentrations, we calculated the excess of NO3 relative with PO4 (N^*^ = NO3 − 16PO4) and the excess of Si relative to NO3 (Si^*^ = Si − NO3). Net Primary Production (NPP, mg.C.m-2.d-1) estimated based on the Standard VGPM algorithm results derived from MODIS AQUA observations between 2003 and 2022 was also used for post-modeling analysis and arranged on the monthly 1° × 1° raster layer of the World Ocean Atlas.

### Biogeographical analyses

First, we analysed the ubiquity and abundance distribution of the 245 lineages (most of them corresponding to species) to highlight ubiquity patterns across dinoflagellate trophic strategies. Prior to the analyses, the species abundances matrix was normalized by dividing the number of reads of species by the total number or reads in the sample, and then transformed using the Hellinger method [45]. Species were compared according to: (i) their total (read) abundance with respect to the 895 sites, (ii) their relative occupancy (the number of sites in which the species is present) and (iii) a Pielou’s evenness index, which indicates how even the distribution of species’ abundance is between sites (see Fig. S2, Table S3).

To cluster the sampling sites according to their environmental conditions, a principal component analysis (PCA) was performed on the 11 environmental annually averaged variables gathered for the 895 sites (SST, SSS, NO3, PO4, Si, MLD, PAR, O2, Sistar, Nstar, and Chla). Then, a hierarchical principal component classification was performed on the PCA scores (see Table S4, Fig. S5).

The biogeographic distribution of dinoflagellate species and their trophic strategies was described thanks to a canonical redundancy analysis (RDA). The target input matrix is the relative species abundance matrix (245 species × 895 sites) and the explanatory matrix corresponds to the matrix of 11 environmental variables, which was centered and standardized. To reduce the dimensionality of the input matrix, 90 species that contributed most to community variance were selected using Escoufier's equivalent vectors method based on a 90% similarity threshold [46]. The nine following variables were selected according to an AIC-based parsimony criterion to retain the variables that significantly constrained the model of the RDA: SST, Chlorophyll a, O2, Si^*^, PO4, MLD, SSS, and PAR (see Table S6).

### Species distribution modelling

#### Selection of species and environmental predictors

Modelling was performed on 72 annotated species that showed sufficient occurrence data (n = 20): 41 mixotrophs (37 CM, 2 pSNCM, 1 eSNCM), 11 strict phototrophs, and 20 strict phagotrophs. Predictors collinearity was inspected through pairwise Spearman correlation coefficients: if two variables presented a coefficient > 0.7, the most ecologically relevant variable was kept. Preliminary models were also run to determine variable importance, then used for predictors selection (Document S7). Finally, species-specific sets of predictors were defined (see Table S8, Fig. S9).

#### Selection of algorithms

Modelling was achieved using the “biomod2” R package [47] which allows to build model ensembles from diverse algorithms depending on their performance. Based on the literature, we chose the following modelling algorithms: Generalized Linear Models, Generalized Additive Models, Multivariate Adaptive Regression Splines, and Artificial Neural Networks.

#### Construction of species distribution models and maps

By combining occurrence data and environmental predictors, SDMs were built allowing us to estimate a habitat suitability index (hereafter HSI) across geographical space for each species. The calibration of the models was carried out using presence/absence data, all the sites where the considered species was not found according to metabarcoding sampling were considered like absences. The parameters of the SDMs were chosen to reduce model complexity and avoid overfitting spurious relationships ([48, 49]; Document S7 for details). A cross-validation approach was adopted followed by a model selection with Jaccard index [50] (see Document S7). In total, 1800 individual models were trained (72 species × 5 algorithms × 5 cross-validations). 872 individual models were retained according to their performance metric (Jaccard index >0.3, see Fig. S10, Fig. S11). These models were then projected on the 12-monthly fields of the predictors covering the full cell grid of the global ocean. Finally, the monthly species-level HSI maps were stacked to create monthly SDM-specific community matrices. From those, the ensemble maps of mean annual HSI were computed for the three trophic strategies.

#### Analysis

Differences in HSI distribution between trophic strategies were evaluated by computing Spearman correlation coefficients between the strategy-specific maps of mean annual HSI. Correlations were also computed between latitudinal HSI profiles and latitude values (northward: from 0° to 82.5°; southward: from 0° to −77.5°) to examine the latitudinal trends in HSI. The mean annual HSI of the trophic strategies were matched against the mean annual values of the 13 environmental variables and a PCA was carried out on these combined variables to compare how these strategies respond to environmental gradients globally (see Fig. S12). The same method was used for species-level HSI projections to examine how strong inter-species differences are within the trophic strategies (see Fig. S13). To investigate regional differences in the mean annual HSI of the trophic strategies, the average HSI of each trophic strategy was calculated by averaging HSI values within the whole surface of the oceanic phytoplankton biomes [51], ensuring a consistent biotic partitioning of the open ocean (see Table S14). The standard-deviation of the species-levels HSI maps was computed within each phytoplankton biome to highlight the regions where species-specific disagreement within trophic strategies is the strongest and/or lowest (see Fig. S15). Details on models outputs are provided in Fig. S16, Fig. S17 and Fig. S18; further information about the dataset and workflow in Fig. S19 and Fig. S20.

## Results

### Trophic specialisation or flexibility has no effect on species ubiquity and evenness

**Figure 1:**
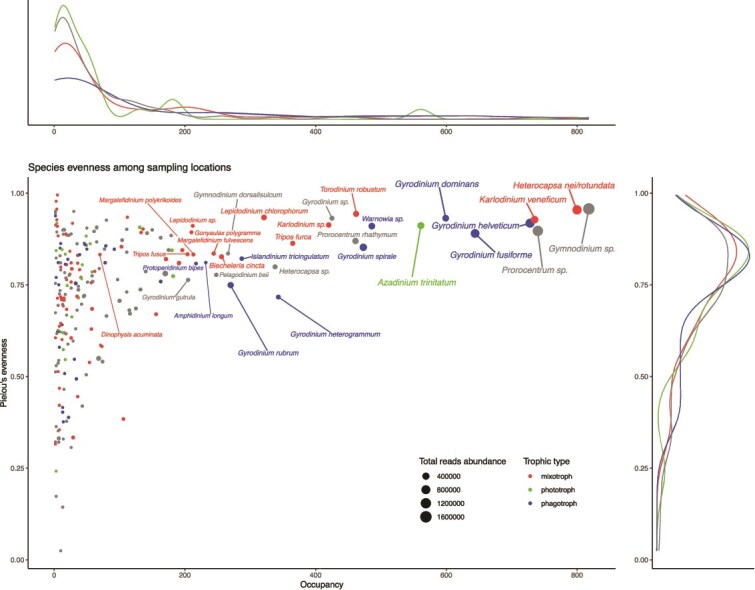
Ubiquity of the studied dinoflagellates species across sampling sites. Each circle represents a species and its radius is proportional to the total count of reads across all samples. The x axis represents the occupancy rate of the species (i.e. the number of unique sites at which it was found). Species displaying high occupancy rate are considered as “ubiquist” whereas the ones displaying low occupancy rates are considered “endemic” species. The y axis represents the Pielou’s evenness index which indicates the abundance profile of a species for each species across the sampling sites. Species with high evenness are equally distributed across sites, whereas species with low are unevenly distributed across sites. The name of the species displaying an occupancy >200 sites are indicated. All the labeled mixotrophic species are CM. *Dinophysis acuminata* is also labeled as a reference [pSNCM]).

The relative ecological success of 245 dinoflagellate species was examined based on global patterns of ubiquity, dominance, and global relative abundance of the ASVs reads across 895 sites (Fig. 1). In general, the total abundance of reads per species increases with occupancy (x-axis; rho = 0.89, p (p-value) = 4.95e-84). The average abundance for each species across sampling sites ranges from 0.05 (*Unruhdinium jiulongensis*, present in 1 site) to 1 603 115 reads (*Gymnodinium* genus, present in 818 sites), with an overall mean abundance of 42 259 reads. Hierarchical clustering differentiated three categories of species based on their evenness and occupancy values (Fig. S2). The first category corresponds to ubiquitous species (i.e. occupancy >473 sites; n (species) = 9) which are homogeneously abundant among sites (i.e. high evenness, mean = 0.92, sd = 0.03, global abundance >453 023 reads). This group thus identifies the dominant species (e.g. *Karlodinium veneficum*, *Gyrodinium fusiforme*). The second category corresponds to rare species (occupancy <106; n = 59) showing relatively low total number of reads (between 206 and 16 119) and low evenness (mean = 0.48, sd = 0.14; e.g. *Gonyaulax cochlea*, *Tripos concilians*). These species are dominant at a few sites, potentially reflecting bloom events occurring at the time of sampling. Finally, the third category corresponds to intermediate species (n = 177) whose occupancy is quite variable (between 1 and 486 sites; mean = 79, sd = 103) and whose evenness is relatively high (between 0.66 and 0.99, mean = 0.80, sd = 0.08). The overall abundance of these species is relatively low, potentially reflecting species that are homogeneously present yet not dominant throughout the oceans (e.g. *Paulsenella vonstoschii*, *Yihiella yeosuensis*).

We could assign a trophic strategy to 56% of the 245 species, resulting in 76 mixotrophic species, 23 strict phototrophic species and 39 strict phagotrophic species (Table S8). 107 species remained trophically unassigned. Among the 76 mixotrophs, 64 are CMs, 7 are eSNCM and 5 are pSNCM, whereas no GNCM was reported. The trophic strategies’ mean occupancy values range between 67, sd = 119 (for strict phototrophs) and 131, sd = 200 (for strict phagotrophs). No significant difference in occupancy distribution was found between trophic strategies (Fig. 1; Kruskal-Wallis test, *P* = 0.86). Mean evenness, like occupancy, appears uniform (between 0.75 for mixotrophs and phototrophs and 0.74 for phagotrophs) across each trophic strategy with no significant differences found between in evenness distributions (Kruskal-Wallis test, *P* = 0.92). Species without any trophic annotation include some abundant and ubiquitous genera like *Gymnodinium*, *Prorocentrum*, *Gyrodinium*, and they display a similar profile as the annotated species. CM (n = 64) display the widest distribution in terms of occupancy (from 1 to 800 sites) and evenness (0.19 to 0.99). eSNCM and pSNCM species (n = 12) display less variable occupancy values (respectively from 1 to 19 and from 1 to 70) and evenness (respectively from 0.20 to 0.70). Yet, eSNCM and pSNCM species do not appear as endemic lineages.

### Few species show contrasted biogeography

**Figure 2:**
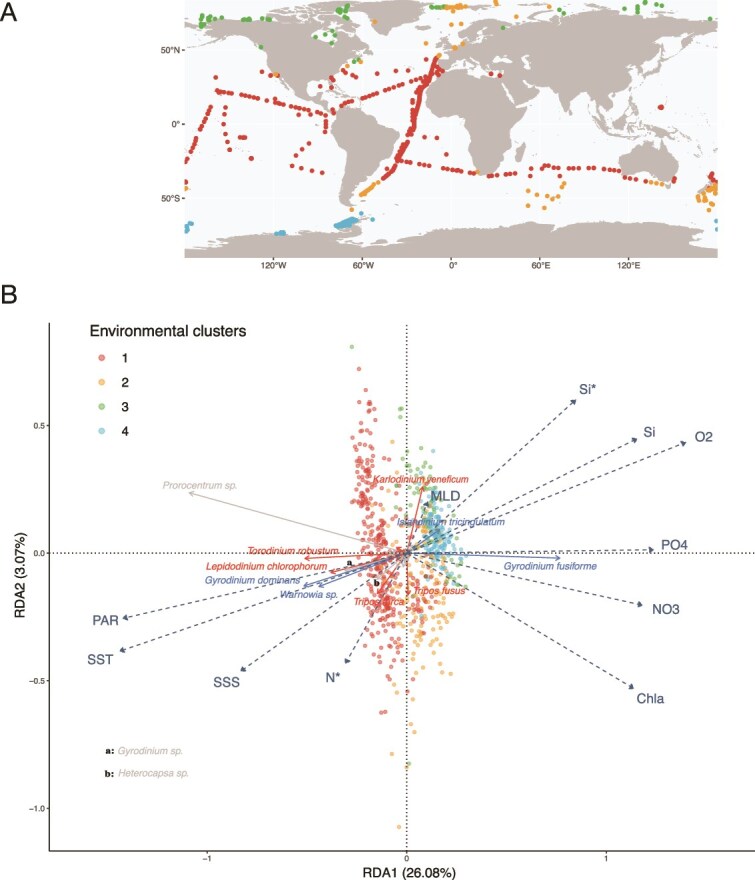
Covariance between environmental gradients and dinoflagellates community structure across their trophic strategy. (A) Spatial distribution of the clusters emerging from the hierarchical clustering performed on a parsimonious environmental space obtained through a PCA computed on the 11 mean annual values of the environmental predictors fitted to the 895 sampling sites (i.e. one point per site). (B) RDA based on nine mean annual environmental predictors as explanatory data and dinoflagellate ASVs abundance matrix as response data, computed on 90 most significant lineages previously selected by Escoufier’s equivalent vectors method. Each point corresponds to a site, colored according to the environmental clustering from PCA computed on same environmental data. Dashed dark blue arrows correspond to the environmental predictors. Plain arrows correspond to selected species whose relative abundance contributes the most to variance explanation. SST: sea surface temperature (°C), chla: surface chlorophyll-a concentration (mg.m-3), si: surface silicates concentration (μmol kg-1), MLD: Mixed layer depth (m), po4: surface phosphates concentration (μmol kg-1), Sistar: (Si^*^) excess of silicates relative to nitrates, PAR: photosynthetically active radiation (μmol m-2.s-1).

The biogeography of *in situ* dinoflagellates abundance related to environmental variables was first studied through multivariate analyses (Fig. 2): the two first components of a PCA based on environmental parameters clearly distinguished four environmental clusters (73.60% of variance explained; Fig. S5). These clusters represent broad oceanic regions (Fig. 2A) that align with a latitudinal gradient primarily influenced by temperature. The red cluster encompasses open-ocean low-latitude sites, while polar regions are represented by the green and blue clusters for the Arctic and Antarctic, respectively. The orange cluster corresponds to temperate or transitional regions that occasionally overlap with the green cluster.

Second, a RDA (Fig. 2B) was performed. The first two axes of the RDA explain 29.15% of the variance, with an overall adjusted R^2^ of 0.32. Backward variable selection based on the AIC resulted in a final model including 11 environmental variables: SST, SSS, chlorophyll-a, O2, Si, Si^*^, N^*^, PO4, MLD, SSS and PAR. The first RDA axis mimics the trends from the first axis of the PCA and separates the eutrophic sites from cold and productive waters (RDA1 > 0) from those sites characterized by warmer, sunnier, and relatively saltier and more oligotrophic waters (RDA1 < 0). The second axis separates the sites from nutrient-rich waters (RDA2 > 0) from sites characterized by high concentrations of Chlorophyll a (RDA2 < 0). Out of the 90 species analysed, 12 show high scores along the first axis (Fig. 2A and Table S6) and only eight of them correspond to those “ubiquitous” species previously defined (Fig. 1).

Finally, the major part of species is located near the center of the RDA which indicates that they do not strongly respond to nutrient gradients. Moreover, as only 12 species show contrasted biogeographies, inferences with the RDA remained qualitative: CM and strict phagotrophs show affinities for oligotrophic waters, but one species of strict phagotrophs (*Gyrodinium dominans*) seems the most successful in polar, eutrophic waters.

### Strict phagotrophy differentiates along latitudinal and regional gradients

**Figure 3:**
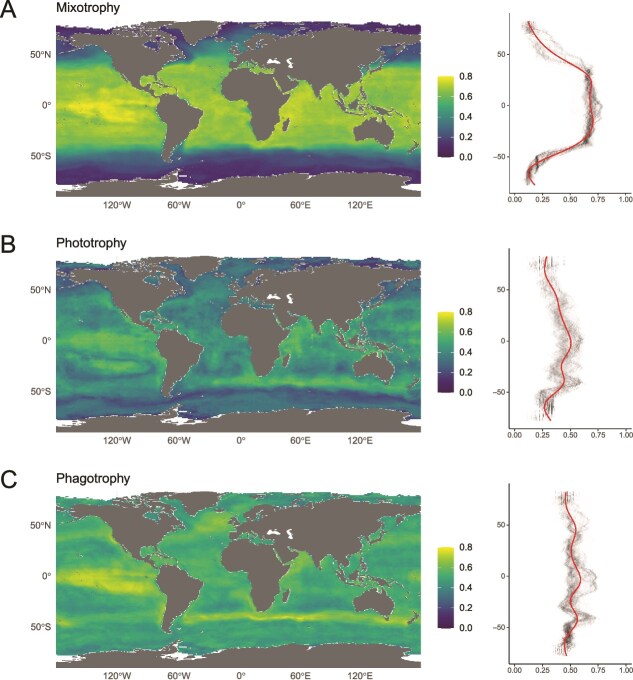
Habitat suitability of trophic strategies across global ocean. (A) Map of annual mean HSI of mixotrophic species. HSI is built by averaging binary HSI for each mixotrophic species. (B) Map of annual mean HSI of strict phototrophic species. (C) Map of annual mean HSI of strict phagotrophic species. Latitudinal density of the HSI for the three maps, respectively mixotrophic, strict phototrophic and strict phagotrophic is displayed on the right of each maps.

The three trophic strategies display different latitudinal profiles of mean annual HSI (Fig. 3). Mixotrophy shows a strongly structured pattern with a high HSI within tropical and sub-tropical regions, whereas latitudinal patterns for the two other strategies are less distinct. Furthermore, spatial distributions show higher similarity between mixotrophy and strict phototrophy (Spearman’s rank correlation coefficients, rho = 0.79; *P* < 2.2e-16) while the distributions are less similar between mixotrophy and strict phagotrophy (rho = 0.52; *P* < 2.2e-16) or more intermediate between strict phagotrophy and strict phototrophy (rho = 0.67; *P* < 2.2e-16). Moreover, the latitudinal profile of decreasing HSI towards the poles is contrasted between trophic groups. For mixotrophy, this decrease strongly correlates with absolute latitude in both hemispheres (northward rho = 0.88, *P* < 2.2e-16; southward rho = 0.94, *P* < 2.2e-16), for strict phototrophy, this decrease is weaker but yet consistent with absolute latitude (northward rho = 0.79, *P* < 2.2e-16; southward rho = 0.71; *P* < 2.2e-16). This decrease is however weaker for strict phagotrophy (northward rho = 0.43, *P* < 2.2e-16; southward rho = 0.49; *P* < 2.2e-16). The highest HSI values (> 0.7) for mixotrophy are restricted to tropical to temperate oceanic regions while the lowest values are found in subpolar and polar environments (Fig. 3A). On the other hand, strict phagotrophy shows HSI values >0.7 within the pacific equatorial upwelling and, to a lesser extent, in the southern Atlantic and Indian ocean transition regions and the temperate/subpolar north Atlantic (Figs. 3C). Conversely, the Pacific, Atlantic and Indian oceanic gyres appear less favourable for strict phagotrophs, as well as some polar regions in the Austral Ocean. Yet, polar regions are globally more suitable for strict phagotrophy (HSI ~ 0.5) compared to the two other trophic strategies (HSI < 0.3) (Fig. 3A, B, C). Strict phototrophy appears as an intermediate case as its highest values (HSI ~ 0.67) are found within tropical and southern transition regions, but to a lesser extent than mixotrophy.

**Figure 4:**
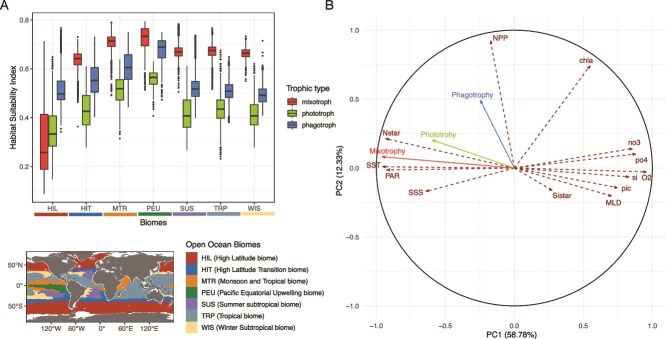
Regional preferences of trophic strategies across open ocean. (A) Boxplot of mean relative HSI distribution within seven open ocean biomes from Hofmann-Elizondo et al. (2021) for the three trophic strategies mixotrophy, strict phototrophy and strict phagotrophy. Every distributions within a biome are significantly different, with global geographic visualisation of the open ocean biomes used for regional partitioning from Hofmann-Elizondo et al. (2021). (B) Principal component analysis computed on global open ocean environmental space (dashed arrows) with mean habitat suitability index of each trophic strategies (plain arrows). The PCA was performed on 13 environmental variables. The modeled HSI of the three trophic were included in the PCA as supplementary quantitative variables. A correlation circle including both variables and individuals is displayed on Fig. S12. SST: Sea surface temperature (°C), chla: surface chlorophyll-a concentration (mg.m-3), si: surface silicates concentration (μmol.kg-1), MLD: mixed layer depth (m), po4: surface phosphates concentration (μmol.kg-1), Sistar: (Si^*^) excess of silicates relative to nitrates, PAR: photosynthetically active radiation (μmol.m-2.s-1).

The biome-level analysis confirms the patterns described above and illustrates the finer regional inter-group differences in mean annual HSI (Fig. 4; Table S14). Strict phagotrophy emerges as the most favourable trait in the High Latitude biome (HIL), which exhibits the broadest HSI distribution. Conversely, all other biomes (tropical and subtropical gyres WIS, TRP, SUS and MTR, and the transition biome HIT) are more favourable to mixotrophy followed by strict phagotrophy. The Pacific Equatorial Upwelling (PEU) region is characterized by the highest HSI scores for the three trophic strategies, with mixotrophy showing the highest values in HSI.

Distribution of trophic strategies within environmental space (Fig. 4A) shows that latitudinal and regional partitioning are well explained by environmental gradients. PC1 (73.33% of total variance) illustrates latitudinal gradient mostly explained by surface temperature and nutrient regimes: negative values along PC1 correspond to oligotrophic, stratified, warm and well-lit environments whereas positive values correspond to eutrophic nutrient-loaded cold and light-limited environments. PC2 (19.56% of variance explained) stands for NPP and phytoplankton biomass gradients. Mixotrophy and to a lesser extent strict phototrophy’s annually averaged HSI are mostly driven by PC1, which is consistent with their latitudinal distribution. They display negative scores, indicating a covariation with variables typical of oligotrophic ocean: high SST, PAR and low nutrients. Strict phagotrophy differentiates by covarying mostly with the primary production gradient which characterizes the PC2, with positive value along this axis. This suggests the affinity of averaged strict phagotrophy for productive environments, with available organic matters to consume.

### Strict phagotrophic species show the widest distribution in the environmental space

**Figure 5:**
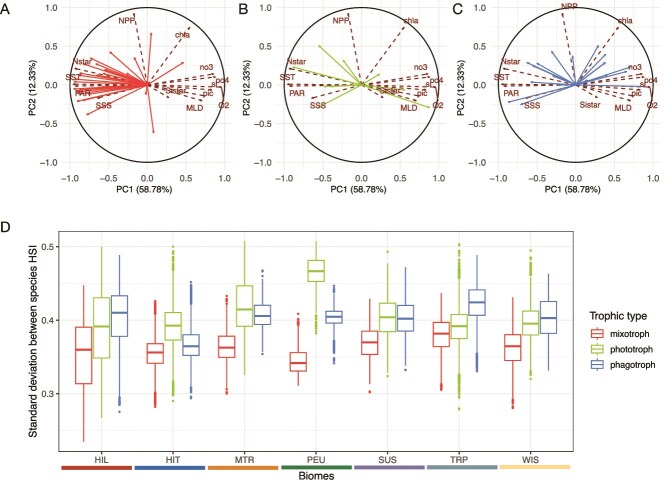
Intra-group variability of responses toward abiotic environments and regional partitioning of standard deviation between species suitability among trophic groups. Principal component analysis computed on global open ocean environmental space (dashed arrows) with mean habitat suitability index of each (A) mixotrophic, (B) strict phototrophic, and (C) strict phagotrophic species (plain arrows) included in the corresponding averaged trait. The PCA was performed on 13 environmental variables. The modeled HSI of the species within trophic groups were included in the PCA as supplementary quantitative variables. A correlation circle including both variables and individuals is displayed on Fig. S12. Species names are displayed on Fig. S13. (D) Boxplot of standard deviation values between HSI of every species representing averaged trophic traits within the seven open ocean biomes from Hofmann-Elizondo et al. (2021). The biomes are the same as those mapped on Fig. 4A. SST: Sea surface temperature (°C), chla: surface chlorophyll-a concentration (mg.m-3), si: surface silicates concentration (μmol.kg-1), MLD: mixed layer depth (m), po4: surface phosphates concentration (μmol.kg-1), Sistar: (Si^*^) excess of silicates relative to nitrates, PAR: photosynthetically active radiation (μmol.m-2.s-1).

The standard deviation across the species’ mean HSI was examined in environmental space for all trophic strategies and each phytoplankton biome to measure the level of intra-group variability (Fig. 5). The standard-deviation between all the HSI values within traits is the highest for strict phagotrophs (mean = 0.41), followed by strict phototrophs (mean = 0.39) and mixotrophs (mean = 0.35; see Fig. S15; significantly different distributions according to Wilcoxon test). On a global average, strict phagotrophy showed contrasted mean HSI patterns compared to mixotrophy and strict phototrophy (Fig. 4B) but our detailed analysis of species-level HSI patterns reveals nuanced patterns (Fig. 5A–C). Mixotrophic species (27 CM, 1 pSNCM) show consistent results with mean mixotrophy HSI (Fig. 5A) as the majority of their distributions covaries positively with higher temperature, nutrient-depleted environments (PCA1 < 0), even if a few species show divergent trends (see Fig. S13A). Overall, mixotrophs show the lowest level of intra-group variability. Phototrophs display a slightly larger intra-group variability (Fig. 5B) with a few species (see Fig. S13C) showing affinity for eutrophic environments (PCA1 > 0). Finally, strict phagotrophy displays the strongest intra-group variability (Fig. 5C). Indeed, the strict phagotrophic species are equitably represented along the PCA1, suggesting a range of affinity between eutrophic (PCA1 > 0) and oligotrophic (PCA < 0) environments. Some strict phagotrophs display an intermediate position, mostly due to their high global mean HSI (*Gyrodinium fusiforme*, *Gyrodinium helveticum*, defined as ubiquist on Fig. 1) or conversely their very spatially restricted high HSI distribution (*Protoperidinium punctulatum*, *Polykrikos kofoidii*, defined as rare).

The standard deviation is the most constant for strict phagotrophic species among oceanic biomes (Fig. 5D), with a minimum found in the HIT region (regional mean = 0.36 ± 0.02) and a maximum in the TRP region (regional mean = 0.42 ± 0.03). Conversely, mixotrophic and strict phototrophic species display greater intra-group variability across the open-ocean biomes, with lower values of intra-group variability for mixotrophy (regional mean ranging between 0.30 and 0.37) than for strict phototrophy (regional mean ranging between 0.33 and 0.46). Notably, the PEU region exhibits the highest intra-group variability among strict phototrophic species, indicating a heterogeneous response of this trophic strategy to this environment while mixotrophic species show the lowest variability, reflecting a more consistent response to this nutrient-rich tropical environment.

## Discussion

On a global scale, trophic strategies exhibit distinct latitudinal patterns of habitat suitability: mixotrophy and strict phototrophy are favoured at low latitudes, while strict phagotrophy displays a weak latitudinal gradient, with suitable habitats found both in and outside the tropics. Such decrease in species habitat suitability towards the poles is consistent with previous studies that modelled dinoflagellates diversity patterns as a unique functional group [38], and correspond to the trend from most planktonic groups [6, 52, 53]. This profile is mainly driven by sea surface temperature, which is the most important predictor of dinoflagellate biogeography whatever their trophic strategies (Fig. S9). The habitat suitability of strict phagotrophy shows a less contrasted profile, suggesting the relative importance of other factors, like nutrient concentration or more likely the presence of preys. Consistently, biogeographical studies of planktonic functional groups based on SDMs highlighted a weak latitudinal HSI gradient for coccolithophores [38], photosynthetic protists which can be phagotrophic during their haploid phase [54, 55]. Coccolithophores and strict phagotrophic dinoflagellates could then be less dependent on surface temperature than other planktonic functional groups, which results for both groups in high HSI in the southern subpolar ocean. However, coccolithophores display more affinity for oligotrophic gyres, suggesting different affinity for nutrient and/or productivity regimes.

Variations in habitat suitability between trophic strategies are also evident at regional scales, primarily driven by resource gradients. Resources like nutrients concentration play a key role in shaping phototrophic protist communities (measures in mesocosms [56]; mixotrophic growth experiments [57]; in-silico predictions [58]), while resources like preys are on the other hand essential for heterotrophic growth [59, 60]. Here, mixotrophy and strict phototrophy are favoured within warm, stratified and oligotrophic environments, i.e. from tropical to temperate regions of the open-ocean. Their HSI is shown to be firstly driven by a nutrient gradient, and secondarily by a productivity gradient. Strict phagotrophy displays the opposite pattern with higher suitability in high latitude biomes with seasonal regimes and within tropical upwelling regions, characterized by high net primary productivity. Strictly phagotrophic species are more favoured in regions where available organic matter from lower trophic levels is more abundant, as they need prey to survive and grow [59, 60]. Interestingly, HSI patterns of strict phagotrophic dinoflagellates align with modeled abundance of shallow (0-200 m) heterotrophic prokaryotes abundance [61], suggesting common distribution drivers like net primary production, which distribution also matches the latter two.

Theoretically, divergent spatial patterns between trophic generalists and specialists would have been expected because of the capacity of the former to switch between organic and inorganic resource uptake, allowing them to outcompete in resources-poor environments [32]. Ward [31] predicted that mixotrophy is advantageous when resource encounter rates are low due to the time required to process nutrients between each prey encounter. Thus, in oligotrophic waters, mixotrophs could ingest as much resources as trophic specialists. Maintenance of a generalist trophic trade-off has been justified by a conservative bet-hedging strategy [32]: i.e. mixotrophs compensate for the risk of depending on a single resource at the individual level, a fitness low-risk strategy regardless of environmental conditions. Edwards [20] also concludes that nutrient-scarcity favours mixotrophs over strict phototrophs and higher irradiance favours mixotrophs over strict phagotrophs. Moreover, the shallower MLD characterising oligotrophic waters should favour mixotrophy due to the vertical separation of resources needed to photosynthesize [62, 63]. Field-based and laboratory-based studies confirmed that mixotrophic protists could account for more than 40% of bacterivory in temperate Atlantic waters and for 37 to 70% in tropical Atlantic waters [64]. Mixotrophic protists were also found to be important grazers in oligotrophic regions such as the subtropical North Pacific [65] and the Mediterranean Sea [66]. Therefore, our results based on empirical macroecological models are in line with both trait-based models as well as *in situ* studies that demonstrated the affinity of mixotrophic species for nutrient-depleted environments. The high HSI observed for mixotrophs in the Pacific equatorial upwelling nuances these findings and indicates that mixotrophy can also thrive and coexist with other trophic strategies in nutrient-rich regions, possibly due to high light irradiance favouring mixotrophs over strict phagotrophs [33].

Conversely, in non-oligotrophic environments, trophic specialists have been theorized as being able to outcompete mixotrophy thanks to their higher growth rates and metabolism that is less expensive to maintain [31]. Specialists would thus be better adapted to high latitude biomes, characterized by higher nutrient concentrations and productivity. Strict phagotrophic dinoflagellates have indeed been observed seasonally as abundant in sub-polar environments [67, 68], and some studies highlight their important grazing role within eutrophic waters [69]. For strict phagotrophs, productivity rather than nutrients is the main suitability driver, even if those two conditions could be met in EBUS or subpolar waters. Strict phototrophy should theoretically be advantageous in a nutrient-rich environment, but our work emphasizes that their HSI is closer to mixotrophs, i.e. favoured in oligotrophic environments. This trend might be explained by the unfavourable seasonally light-limiting conditions in high latitude biomes. Furthermore, when comparing with distribution patterns of diatoms (strict phototrophic protists), HSI partly follows species richness patterns, peaking in productive low-latitude upwellings [70]. However, unlike Busseni’s findings, richness in oligotrophic gyres is high, and increase in subpolar/polar regions is less pronounced. In this regard, our patterns for strict phototrophs are closer from diatoms species richness distribution described in [38] with a clear latitudinal decrease of richness toward high latitudes.

We observed that distribution patterns of trophic traits differentiate well mixotrophy, a generalist strategy, from strict phagotrophy, a specialist strategy. This trend is based on average trophic traits responses along abiotic gradients, meanwhile strict phototrophy shows a response close to mixotrophy. When looking at species responses within traits, two patterns emerge: mixotrophic species overall show consistent responses toward abiotic gradients, while strict phagotrophic species display various affinities. Several explanations could be invoked: (i) The inaccuracy of trophic annotation: the overlapping patterns observed between strict phototrophic and mixotrophic species could suggest that strict phototrophs actually are unnoticed CM due to the difficulty to observe phagotrophy in cultures or even natural populations [71, 72]. Trophic annotation is indeed likely to carry a bias toward cultured species or influenced by the context of trait observation. Depending on obligate or facultative mixotrophs, but also the spectrum of mixotrophic strategies described above, the reliance on phagotrophy or phototrophy is highly variable across taxa and environmental context [24, 25]; (ii) trophic specialists could result of independent trait losses from a generalist ancestor (e.g. phagotrophy or phototrophy losses [73, 74], which occurred in different environmental contexts, explaining the niche differences among species carrying the same trait); (iii) other factors or functional traits could influence these responses toward abiotic gradients.

Among those traits, cell size is likely to contribute to the divergent biogeographical patterns between species. Indeed, smaller cells are more efficient in nutrient acquisition due to higher surface/volume ratio, enabling a better diffusion through their membrane at low nutrient concentrations [75] and thus maintaining positive growth rates in such conditions [76]. Theoretical models thus predict that small cells are favoured in oligotrophic regions of the open ocean, like subtropical gyres, as they can maintain low inorganic resource concentrations [32]. Conversely, in resource-replete regions of the ocean like subpolar gyres, high nutrient concentrations enable small cells to grow but they are subject to predation by larger cells, which helps maintain relatively high nutrient levels. These modelling results are consistent with trophic distributions discussed above; mixotrophic species with better affinity to nutrient-depleted, intensely lit and highly stratified oligotrophic regions could be smaller due to limited growth rates. This preference stems from their optimal utilisation of both phototrophy and phagotrophy compared to specialists. Conversely, specialists should have larger cells in the more productive conditions as the latter provide them with sufficient prey concentrations to maintain strong growth rates allowing them to outcompete generalist strategies. This possible competitive exclusion mechanism was verified for other microalgae, strict phototrophic Chrysophytes, which displayed bigger cells and bigger genomes than mixotrophic ones, whereas strict phagotrophic Chrysophytes however showed smaller genomes and cell sizes [77]. Size differences are also invoked to explain coexistence of trophic strategies under a range of conditions [78], as in high phosphors and light supplies environments [79], which could also explain the high HSI of the three trophic strategies in the pacific upwelling region.

As trophic strategies do not appear here to be a discriminating factor in explaining patterns of dominance or equitability among epipelagic dinoflagellate communities, it is likely that differences in other life history traits (e.g. reproductive strategy, growth rate, cyst formation) and/or biotic interactions have an influence. As an example, the pSNCM species *Dinophysis acuminata* is found among the “intermediate” species which could be explained by its obligate interaction (kleptoplasty here) with its ciliate preys belonging to the *Mesodinium* species [80]. Such obligatory biotic interaction restrains the niche of *D. acuminata* but it cannot be accurately represented by solely labelling the species as a pSNCM. Additionally, scale-dependent processes could be invoked to explain abundance-related community analysis and SDMs outputs differences. Indeed, abundance even more than occurrence is likely to carry the imprint of biotic factors and local dispersal [81]. Therefore, relative abundance patterns of Dinoflagellates could be influenced by both interactions (competition, predation) and mesoscale hydrodynamic activity (e.g. eddys) while occurrence patterns retrieved at global scale should be more influenced by abiotic environmental factors [82], like the predictors used here to train the SDMs. Integrating abundance as the explained variable of the models would be however challenging due to the numerous gene copies in dinoflagellates, resulting in a biased correspondence between reads and cells absolute number [10, 83, 84]. Once again, even if global trends differentiate trophic strategies’ habitat suitability, divergent patterns of ubiquity and distribution profiles between species within trophic traits, suggest the importance of diversification and niche occupation, depending on species-specific’s physiological optima.

Disentangling the trophic trait biogeography of dinoflagellates is a first step toward a better understanding of the functional diversity underlying ecological and biogeochemical processes. Mixotrophic dinoflagellate species increase the complexity of biotic interactions within planktonic food webs by functioning simultaneously as primary producers and consumers. The contribution of mixotrophy to carbon fluxes and trophic transfer remains poorly understood and only a few models have attempted to quantify its contribution to nutrient acquisition [27]. Identifying the hotspots of trophic groups’ presence then provides relevant insights into the trophic structure of planktonic communities, and the ecosystemic functions they carried out across oceanic regions. For instance, we find that constitutive mixotrophs display congruent responses toward abiotic gradients. Given predictions that oligotrophic gyres will expand [85], this suggests that mixotrophic dinoflagellates may find increasingly suitable habitats in a changing open ocean. On the contrary, the divergent responses observed among dinoflagellates with strict trophic strategies imply more complex responses in the context of global ocean warming and oligotrophization. For example, phagotrophic dinoflagellates colonize a wide range of environments and may show species-specific and different responses to warming and increased stratification, which makes predictions for this group more challenging. Very few studies so far explored the links between trophic strategy and nutrient transfers for mixotrophic (*Prorocentrum cf. balticum*, [14]) and phago-heterotrophic (*G. dominans*, [86]) dinoflagellates. Given the current large knowledge gaps on the topic, this field offers considerable potential for advancing our understanding through future research. Further global-scale studies would be required to quantify the abundance and biomass of dinoflagellates trophic groups to link their ecological success to quantitative contribution to biogeochemical cycles. Omics represent, in this regard, a promising source of data, as previous studies have successfully linked genomic and transcriptomic markers to the abundance or biomass of certain phytoplankton species, including some dinoflagellates [84, 87].

Understanding the biogeography of trophic traits, and particularly the niche consistency between trophic generalists and specialists, offers valuable insights into the ecology and evolution of dinoflagellates. Contrary to expectations, we find that trophic generalists tend to occupy a narrower range of environmental conditions (i.e. warm and oligotrophic habitats from the tropical ocean; Fig. 5A) relative to trophic specialists (Fig. 5B–C). We believe this discrepancy may be linked to multiple independent events of gains or losses of plastids/endosymbionts in dinoflagellates reported by evolutionary studies, and most probably from a mixotrophic ancestor [74]. The fact that phago-heterotrophic dinoflagellates occupy a range of different environments may be due to convergent adaptations from a generalist mixotroph ancestor, coupled with potential competitive contexts [25, 88]. As other specialist species (i.e. strict phototrophs) display similar abiotic affinities than mixotrophs, generalists and specialists may coexist in oligotrophic environments, potentially resulting from sympatric speciations.

Data-driven correlative approaches are powerful to describe emergent spatial patterns in community ecology but they rely on strong assumptions (see Document S7). Ignoring potential biotic interactions when modeling the response of species to their environment is one of them, but is not likely to have a strong impact on our global-scale modeling as discussed above. This question is still intensely debated in the biogeography and community ecology fields and currently arouse promising methodological developments [89–91]. We then encourage future modelling studies about protists trophic traits biogeography to focus on biotic factors shaping planktonic communities, especially in the case of phagotrophy and predation.

Our work paves the way for further data-driven global studies with a stronger concern of the trophic trait influence and its specialisation on species biogeography. Metatranscriptomics stand as a hopeful tool to better describe the expression of the traits of interest [92], which could help, coupled with strong correlative approaches and by circumventing the metabarcoding caveats, to understand better the trophic niche of dinoflagellates and to a larger extent, marine protists.

## Supplementary Material

Fsupplementary_mat_rihm_ismecomm_revised_ycaf153

## Data Availability

The scripts and data underlying this article are available in GitHub, at https://github.com/RihmG/Dino_trophic_biogeo
