# Supplementary material for: Do trophic strategies shape biogeography and environmental niches? Marine dinoflagellates as a case study
Source: ISME Commun. 2025 Sep 16;5(1):ycaf153. doi: 10.1093/ismeco/ycaf153 (PMC12452278; doi:10.1093/ismeco/ycaf153)
Supplement: Fsupplementary_mat_rihm_ismecomm_revised_ycaf153 [file fsupplementary_mat_rihm_ismecomm_revised_ycaf153.pdf]

Supplementary Material

Supplementary Material list

S1: Summary of Dinophyceae metabarcoding dataset.

S2: Species partitioning based on their Pielou's evenness and occupancy across sampling stations

S3: Pairwise Wilcoxon tests results of the influence of trophic types on the distribution of occupancy, evenness, abundance

S4: Table of environmental predictors used for this study.

S5: Cluster analysis plots.

S6: Scores of the 12 most significant species projected on the first (RDA1) and the second (RDA2) axis of the RDA (Fig. 2A).

S7: ODMAP protocol

S8: List of species with the corresponding environmental predictors, used for calibration of individual models

S9: Distribution of the scores of relative variable importance computed for each species during model calibration and summarized for the three trophic types

S10: Response Curves of the 3 trophic types for the 9 environmental predictors used for calibration

S11: Jaccard Scores of individual models

S12: Biplot of the principal component analysis computed on mean habitat suitability (HSI) values of each mixotrophic species, with environmental supplementary variables.

S13: Principal component analysis computed on the open-ocean environmental space, with mean habitat suitability (HSI) values of each modeled species, with environmental supplementary variables as supplementary variables.

S14: Wilcoxon tests for trophic types distribution among open-ocean phytoplankton biomes

S15: Boxplot of standard deviation between HSI of every species representing averaged trophic strategy at the open-ocean scale.

S16: Maps of the standard deviation of individual models results computed for each ensemble models, averaged per monthly projection and per trophic types

S17: Maps of annually averaged habitat suitability index of mixotrophy depending on the modeling technique

S18: Monthly variability of habitat suitability index global distribution for the three trophic types.

S19: Temporal and spatial sampling effort for Dinophyceae ASVs.

S20: Schematic diagram of the workflow carried out in this study. Red squares represent the input data, and blue italic text indicates the analyses performed.

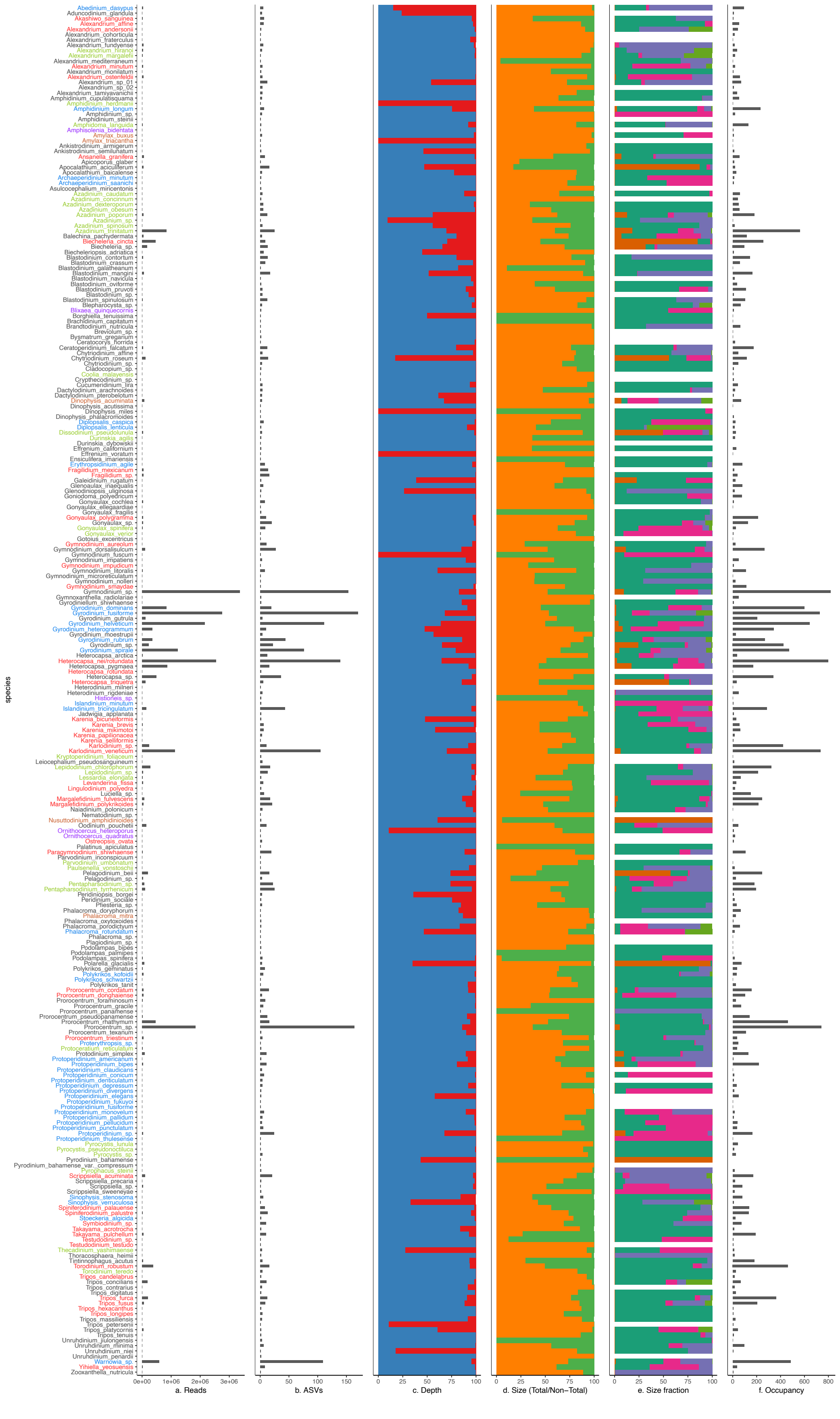

species
0e+00
1e+06
2e+06
3e+06
a. Reads
0
50
100
150
b. ASVs
0
25
50
75
100
c. Depth
0
25
50
75
100
d. Size (Total/Non−Total)
0
25
50
75
100
e. Size fraction
0
200
400
600
800
f. Occupancy

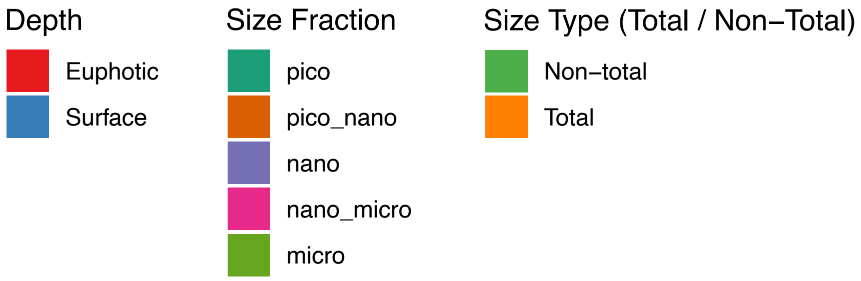

Figure S1: Summary of Dinophyceae metabarcoding dataset. The amplicon sequence variants (ASVs) corresponding to each species of the dataset were summed for comparing their abundance and diversity (columns a and b). Sampling depth ('surface' or 'euphotic') is based on the classification of the metaPR2 database (column c). Size fraction was divided into two columns: (i) Size type: 'Total' and 'Non-total' if the ASV was found in the 'Total' category of the metaPR2 classification or not (column d); and (ii) Size Fraction: 'pico', 'pico-nano', 'nano', 'nano-micro', and 'micro' if the ASV was found in a particular size fraction (column e). The occupancy of the ASV (i.e., the number of unique sites where the ASV was retrieved) is indicated in column f.

The species names are colored according to their trophic strategy: blue for strict phagotrophs, green for strict phototrophs, red for CM, brown for pSNCM and purple for eSNCM.

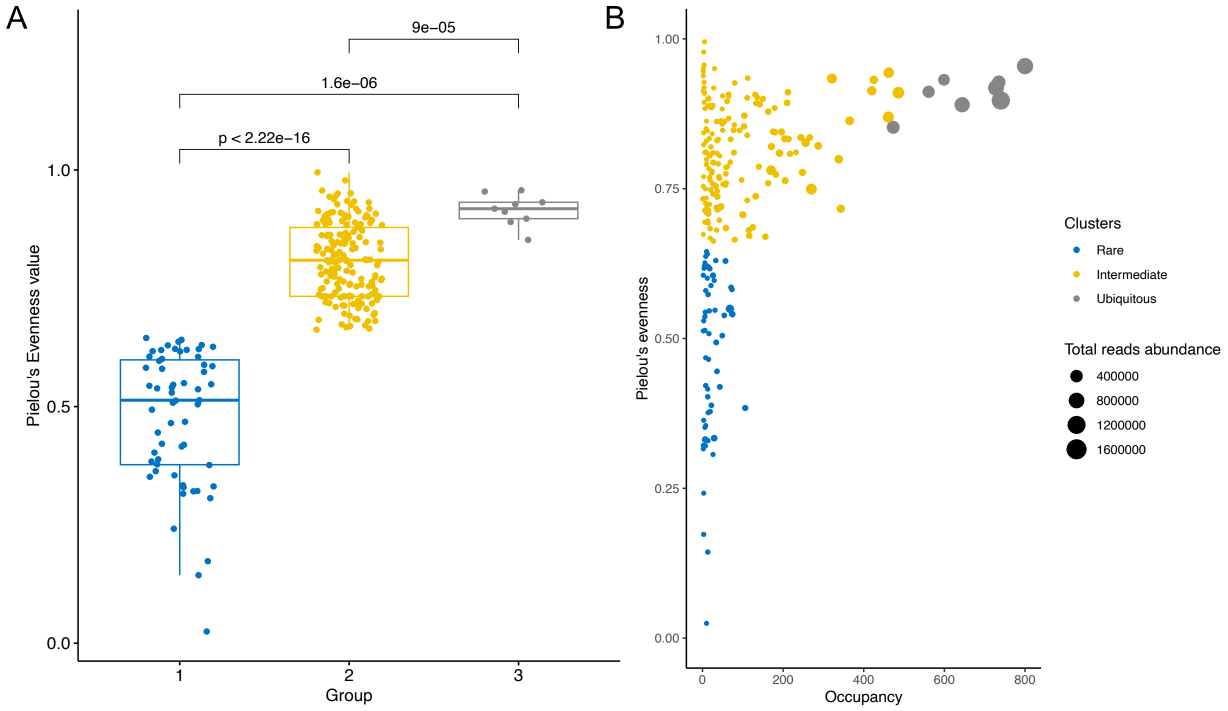

Figure S2: Species partitioning based on their Pielou’s evenness and occupancy across sampling stations. A: Distribution of evenness value of the three clusters. The distributions are significantly different at the 0.05 level according to Wilcoxon tests. B: Evenness profiles of the species depending on their occupancy across sampling stations. The cluster 1 is termed ‘rare' species, the cluster 2 ‘intermediate’ species, and the cluster 3 is termed ‘ubiquitous’ species.

Wilcoxon tests for occupancy distribution between trophic types

.y.	group1	group2	n1	n2	statistic	p	p.adj	p.adj.signif
occupancy	mixotroph	phototroph	75	23	889.500	0.824	1	ns
occupancy	mixotroph	phagotroph	75	39	1,384.500	0.643	1	ns
occupancy	phototroph	phagotroph	23	39	419	0.672	1	ns

Wilcoxon tests for evenness distribution between trophic types

.y.	group1	group2	n1	n2	statistic	p	p.adj	p.adj.signif
Jinvsites	mixotroph	phototroph	69	22	764	0.967	1	ns
Jinvsites	mixotroph	phagotroph	69	36	1,302	0.688	1	ns
Jinvsites	phototroph	phagotroph	22	36	412	0.805	1	ns

Wilcoxon tests for abundance distribution between trophic types

.y.	group1	group2	n1	n2	statistic	p	p.adj	p.adj.signif
Tot_abundance	mixotroph	phototroph	75	23	1,083	0.065	0.196	ns
Tot_abundance	mixotroph	phagotroph	75	39	1,496	0.844	1	ns
Tot_abundance	phototroph	phagotroph	23	39	362	0.212	0.636	ns

Table S3: Pairwise Wilcoxon tests results of the influence of trophic types on the distribution of 1. occupancy, 2. evenness, 3. abundance

Environmental Predictor	Abbreviation	Units	Source	Reference
Sea Surface Temperature	SST	°C	World Ocean Atlas (WOA) 2018	Locarnini MM, Mishonov AV, Baranova OK, Boyer TP, Zweng MM, Garcia HE, Reagan JR, Seidov D, Weathers KW, Paver CR, Smolyar I (2018). World Ocean Atlas 2018, Volume 1: Temperature. Ref. NOAA Atlas NESDIS 81, 52pp.. A. Mishonov Technical Editor. https://archimer.ifremer.fr/doc/00651/76338/
Sea Surface Salinity	SSS	-	World Ocean Atlas (WOA) 2018	Zweng MM, Reagan JR, Seidov D, Boyer TP, Locarnini MM, Garcia HE, Mishonov AV, Baranova OK, Weathers KW, Paver CR, Smolyar I (2019). World Ocean Atlas 2018, Volume 2: Salinity. Ref. NOAA Atlas NESDIS 82, 50pp.. A. Mishonov Technical Editor. https://archimer.ifremer.fr/doc/00651/76339/
Mixed Layer Depth	MLD	m	SODA 3.4.2	Carton, J. A., G. A. Chepurin, and L. Chen, 2018: SODA3: A New Ocean Climate Reanalysis. J. Climate, 31, 6967–6983, https://doi.org/10.1175/JCLI-D-18-0149.1.
Photosynthetically Active Radiation	PAR	$\mu mol.m^{-2}.s^{-1}$	SeaWIFS	NASA Goddard Space Flight Center, Ocean Ecology Laboratory, Ocean Biology Processing Group; (2018): Sea-viewing Wide Field-of-view Sensor (SeaWiFS) Photosynthetically Available Radiation Data, NASA OB.DAAC
Dissolved Oxygen Concentration	O2	$\mu mol.kg^{-1}$	World Ocean Atlas (WOA) 2018	Garcia HE, Weathers KW, Paver CR, Smolyar I, Boyer TP, Locarnini MM, Zweng MM, Mishonov AV, Baranova OK, Seidov D, Reagan JR (2019). World Ocean Atlas 2018, Volume 3: Dissolved Oxygen, Apparent Oxygen Utilization, and Dissolved Oxygen Saturation. Ref. NOAA Atlas NESDIS 83, 38pp.. A. Mishonov Technical Editor. https://archimer.ifremer.fr/doc/00651/76337/
Surface Particulate Inorganic Carbon Concentration	PIC	$mol.m^{-3}$	SeaWIFS	NASA Goddard Space Flight Center, Ocean Ecology Laboratory, Ocean Biology Processing Group; (2018): Sea-viewing Wide Field-of-view Sensor (SeaWiFS) Particulate Inorganic Carbon Data, NASA OB.DAAC.
Surface Chlorophyll-a Concentration	Chla	$mg.m^{-3}$	SeaWIFS	NASA Goddard Space Flight Center, Ocean Ecology Laboratory, Ocean Biology Processing Group; (2018): Sea-viewing Wide Field-of-view Sensor (SeaWiFS) Chlorophyll Data, NASA OB.DAAC.
Surface Nitrates Concentration	NO3	$\mu mol.kg^{-1}$	World Ocean Atlas (WOA) 2018	Garcia HE, Weathers KW, Paver CR, Smolyar I, Boyer TP, Locarnini MM, Zweng MM, Mishonov AV, Baranova OK, Seidov D, Reagan JR (2019). World Ocean Atlas 2018. Vol. 4: Dissolved Inorganic Nutrients (phosphate, nitrate and nitrate+nitrite, silicate). Ref. NOAA Atlas NESDIS 84, 35pp.. A. Mishonov Technical Editor. https://archimer.ifremer.fr/doc/00651/76336/
Surface Phosphates Concentration	PO4	$\mu mol.kg^{-1}$	World Ocean Atlas (WOA) 2018	Garcia HE, Weathers KW, Paver CR, Smolyar I, Boyer TP, Locarnini MM, Zweng MM, Mishonov AV, Baranova OK, Seidov D, Reagan JR (2019). World Ocean Atlas 2018. Vol. 4: Dissolved Inorganic Nutrients (phosphate, nitrate and nitrate+nitrite, silicate). Ref. NOAA Atlas NESDIS 84, 35pp.. A. Mishonov Technical Editor. https://archimer.ifremer.fr/doc/00651/76336/
Surface Silicates Concentration	Si	$\mu mol.kg^{-1}$	World Ocean Atlas (WOA) 2018	Garcia HE, Weathers KW, Paver CR, Smolyar I, Boyer TP, Locarnini MM, Zweng MM, Mishonov AV, Baranova OK, Seidov D, Reagan JR (2019). World Ocean Atlas 2018. Vol. 4: Dissolved Inorganic Nutrients (phosphate, nitrate and nitrate+nitrite, silicate). Ref. NOAA Atlas NESDIS 84, 35pp.. A. Mishonov Technical Editor. https://archimer.ifremer.fr/doc/00651/76336/
Net Primary Production	NPP	$mg.C.m^{-2}.d^{-1}$	Aqua/MODIS VGPM algorithm	Behrenfeld, MJ, PG Falkowski Limnology and Oceanography 1997a, Volume 42: 1-20 Photosynthetic rates derived from satellite-based chlorophyll concentration https://doi.org/10.4319/lo.1997.42.1.0001 Ocean Productivity website http://orca.science.oregonstate.edu/index.php

Table S4: Environmental variables used for multivariate analysis, calibration and extrapolation of SDMs and model outputs analysis (Figures 2-5).

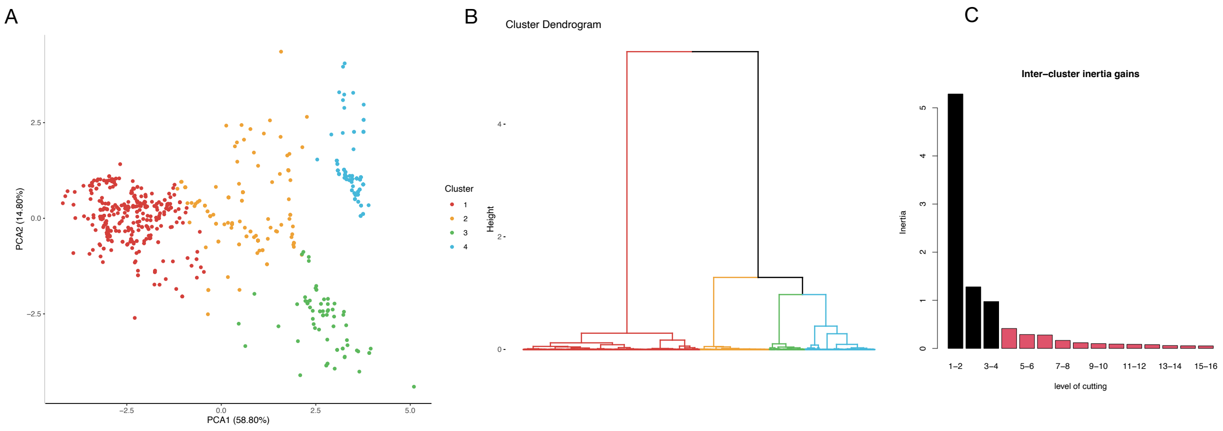

Figure S5: Cluster analysis. A hierarchical classification was first performed on the principal components of the PCA, based on Ward's aggregation criterion. This was consolidated by a k-mean clustering in order to refine the clusters. The four clusters were chosen based on the largest relative drop in inter-cluster inertia, with the transition from three to four clusters still showing a substantial gain in group separation.

A: Individual graph plot for the environmental PCA used to cluster the stations, the colors represent the different clusters based on abiotic environmental variables. B: Dendrogram representing the stations after hierarchical clustering on principal components. C: Barplot of the inter-cluster inertia gain as a function of the number of clusters.

Species	Trophic type	Mixotrophic type	RDA1	RDA2
Lepidodinium chlorophorum	mixotroph	CM	-0.38	-0.08
Torodinium robustum	mixotroph	CM	-0.51	-0.02
Gyrodinium sp.	-	-	-0.31	-0.07
Prorocentrum sp.	-	-	-1.09	0.24
Tripos furca	mixotroph	CM	-0.15	-0.16
Gyrodinium fusiforme	phagotroph	-	0.77	-0.02
Warnowia sp.	phagotroph	-	-0.44	-0.13
Islandinium tricingulatum	phagotroph	-	0.17	0.09
Gyrodinium dominans	phagotroph	-	-0.51	-0.13
Karlodinium veneficum	mixotroph	CM	0.08	0.26
Tripos fusus	mixotroph	CM	0.01	-0.16
Heterocapsa sp.	-	-	-0.15	-0.1

Table S6: Scores of the 12 most significant species projected on the first (RDA1) and the second (RDA2) axis of the RDA (Fig. 2A). Three CM (*Torodinium robustum*, *Lepidodinium chlorophorum*, *Tripos furca*), two strict phagotrophs, (*Gyrodinium dominans* and *Warnowia* sp.), and three trophically unannotated taxa (*Prorocentrum* sp., *Gyrodinium* sp., *Heterocapsa* sp.), are characterising oligotrophic, warm and salty environments (RDA1 < 0). Two strict phagotrophs (*Gyrodinium fusiforme* and *Islandinium tringulatum*) and one CM (*Karlodinium veneficum*), have affinity for eutrophic, cold and productive environments (RDA1 > 0). Three mixotrophic species display the highest scores along RDA2 (*Tripos fusus*, *Tripos furca*, *Karlodinium veneficum*).

Documents S7: ODMAP PROTOCOL

This protocol is a standardized way to report species distribution modeling, proposed by [1].

"Do trophic strategies shape biogeography and environmental niches? Marine dinoflagellates as a case study"

Model objective

Model objectives:

Mapping and interpolation. We model the spatial distribution of the habitat suitability of dinoflagellates species in order to map the distribution of their trophic trait in the global ocean.

Target output:

Map the global mean annual habitat suitability of the three trophic types of Dinophyceae (mixotrophic, strict phagotrophic, strict phototrophic) to investigate the biogeography of trophic traits and highlight the potential coexistence of trophic types within open ocean biomes.

Focal Taxon

Focal Taxon:

Dinoflagellates, that have been reduced to 72 extant Dinophyceae lineages, representative of the three trophic types of interest.

Location

Location: Global surface Ocean,

Scale of Analysis

Spatial extent: Longitude -180, 180; Latitude -90, 90

Spatial resolution:

Occurrences were sampled on an ocean cell grid of 1° x 1° (~110 km at the Equator). This resolution was chosen to match the resolution of the climatologies of the environmental predictors used in the study (i.e., lowest common denominator). Moreover, this resolution helps to remove spurious very-coastal occurrences whose coordinates might be incorrect.

Temporal extent: The occurrence data were obtained by integrating several field surveys, oceanographic cruises and a few time-series, all conducted between 2002 and 2018.

Temporal resolution: To match the biological occurrence data, the environmental predictors used in this study are monthly climatologies calculated from monthly fields representative of the 1955-2018 time period.

Boundary:

Biodiversity data

Observation type: Georeferenced occurrences from metabarcoding surveys from at least 59 datasets gathered in the MetaPR2 database [2].

Response data type: Presences / Absences

Predictors

Predictor types: bioclimatic, habitat

Hypotheses

Hypotheses: see below

Assumptions

Model assumptions: We assumed that (i) species are at equilibrium with their environment, i.e. the populations occupy their whole potential range, (ii) niche conservatism occurs through time, (iii) the spatial distribution of the species modelled are not limited by dispersal, (iv) the environmental abiotic predictors chosen is this study control the environmental niche and the spatial distribution of the species of interest, (v) the 18S metabarcoding sampling allows us to define true absences.

Algorithms

Modelling techniques:

The types of algorithms were chosen to cover the different modeling techniques commonly used in SDM-based studies. This way, we cover a wide range of model complexity that allows us to account for model-choice uncertainty in our diversity predictions. We used regression-based approaches like Generalized Linear Models (GLM), Generalized Additive Models (GAM), Multivariate Adaptive Regression Splines (MARS) and also machine-learning systems, including neural networks (ANN) and Boosted Regression Trees (BRT).

Model complexity: We kept model parametrization as simple as we could.

Model ensembles: All the individual models meeting our validation criteria were stacked by computing arithmetic mean. All individual models were given the same weight as they were already selected by Jaccard metrics.

Model workflow: Species distribution models were trained with occurrence data corresponding to 72 dinoflagellate species that showed at least 20 presences across the study extent. Monthly environmental predictors allowed us to fit individual models as follows: for each species, 1 set of models was built, including for each set 5 algorithms types (GLM, GAM, MARS, ANN, BRT) and 5 folds of cross-validation. This results in the creation of 5 x 5 = 25 individual models per species.

In total, 25 models x 72 species = 1800 individual models were built. Evaluating these models based on the Jaccard index allowed us to select 879 satisfying individual models. Binary projections of habitat suitability maps were created using a threshold maximizing the Jaccard index for each individual model. Stacks of the binary ensemble models by trophic type were then created in order to draw final maps of the habitat suitability of trophic type.

Software

Software: R version 4.2.2 [3] with packages *terra* 1.7-29, [4], *raster* 3.6-11 [5], *biomod2* 4.2-2 [6], *sf* 1.0-9 [7], and others.

Code availability: All scripts are provided on GitHub at (https://github.com/RihmG/Dino_trophic_biogeo)

Data availability:

Data

Biodiversity data

Taxon names: 72 Dinophyceae species, see Supplementary Material for all the species names and their trophic assignment.

Taxonomic reference system: Current names used by metaPR2 and validated against the list of accepted species names from WoRMS and AlgaeBase.

Ecological level: Assemblage of 72 dinoflagellates species modelled independently

Data sources: Occurrence data were downloaded from the metaPR2 database with the following specific filtering: we chose only those ASVs that correspond to the V4 region of the 18S rRNA, regardless of sampling size fraction.

Sampling design: As the occurrence data come from several datasets issued from different projects, there were no specific sampling design for this study. The design of our study thus represents an emergent property stemming from the integration of various and independent field surveys whose design we could not control.

Sample size: After extraction and cleaning of the occurrence data, we obtain a presence/absence matrix of 251 species X 895 unique sites. After re-sampling the occurrence on to the 1° x 1° cell grid, this matrix was reduced to 531 occurrences (absences and presences) in total. A list of candidate species was then established thanks to the number of presences: species that were showing more than 20 presences (after rasterization) were selected, which represents 72 species (35 mixotrophs, 16 strict phototrophs, 21 strict phagotrophs).

Absence Data: As the data structure allowed us to build a presence-absence matrix, all the sites where the species were not found are considered like true absences, assuming that metabarcoding was deep enough during sampling to ascertain true absences at a given place.

Validation data: As independent observations were not available at the scale of our study, data were split for training and validation for variables selection and then for model calibration. For predictor selection, smaller models were trained with 80% of the data and validated with 20% of the data. For model calibration, models were trained with 70% of the data and cross-validated 5 times with 30% the data.

Test data: See above.

Predictor variables

Predictor variables:

The environmental used in this study are the following : Sea Surface Temperature (SST), Sea Surface Salinity (SSS), Mixed Layer Depth (MLD), Photosynthetically Active Radiation (PAR), Dissolved Oxygen concentration (O2), Surface Particulate Inorganic Carbon (PIC), Surface Chlorophyll concentration (Chla), Surface Nitrates concentration (NO3), Surface Phosphates concentration (PO4), Surface Silicates concentration (Si), Excess of nitrates on phosphates (N*), Excess of Silicates to Nitrates (Si*). Those variables were taken from [8] (see table S18). PO4, Si, NO3 and Chla variables were logarithmically transformed prior to the analysis.

Data sources: World Ocean Atlas (WOA) 2018, SODA 3.4.2, SeaWIFS.

Spatial extent: Longitude -180, 180; Latitude -90, 90

Spatial resolution: All predictor variables were aggregated to 1°x1° resolution.

Coordinate reference system: WGS84 (EPSG:4326)

Temporal resolution: Monthly climatologies representative of the 1955-2018 time period.

Data processing:

Model

Variable pre-selection

Variable pre-selection was carried out independently from the species data. For each month, we first chose non colinear variables, using the function *removeCollinearity* in the *virtualspecies* R package [9]. An ascendant hierarchical clustering was performed based on a pairwise distance matrix calculated with Spearman correlation coefficients. A threshold of 0.5 was applied to discriminate variables in clusters, and then a manual selection was made within each cluster based on the biological plausibility and interest of variables. We then obtained a set of non-colinear environmental variables for each month.

After this pre-selection, we calibrated models for each species with monthly data. The prevalence of the biomod function *BIOMOD_Modeling* was set to 0.7 in order to give less weight to absences than presences. Finally, the number of permutations for computing variable importance was set to 10. The variable importance for each individual model was found by calculating a Spearman correlation between the prediction of the model and a randomly re-sampled model. The more different the prediction is from the randomized model, the more the variable is important to explain the variation along the environmental gradient. Here, for each individual model, we kept variables that show a median variable importance > 0.1.

Finally, for each species, the number of predictors were lowered to a number that allow modeling regarding the number of occurrences (number of predictors < or = number of presence/10).

Multicollinearity

See Variable pre-selection above. Pairs of multicollinear variables were not included as predictors in the models.

Model settings

GLM: Type (quadratic), interaction level (0), family (binomial logit), test (AIC), control (epsilon = 1e-08, maxit = 50, trace = FALSE).

GAM: algo (GAM_mgcv), type ('s_smoother'), k (5), interaction.level (0), family (binomial logit), method (GCV.cp), select (FALSE), knots (NULL), paramPen (NULL)

ANN: NbCV (5), size (NULL), decay (NULL), rang (0.1), maxit (200)

MARS: type (simple), interaction.level (0), nk (NULL), penalty (2), thresh (0.001), nprune (NULL), pmethod (backward).

Model extrapolation: Not done

Model estimates

Variable importance: Each predictor was assessed for each month and each species thanks to the variable importance calculation of *biomod2* [6] (*variables_importance* function).

Model selection - model averaging - ensembles

Model selection: Selection of individual models was based on the Jaccard index. A threshold of 0.3 was retained as it allowed us to keep 879 individual models of the 1800 models created (corresponding to 30 mixotrophs, 19 strict phagotrophs and 9 strict phototrophs). After inspection of the models retained, it appeared that some species were more frequently retained than others (e.g., all the models for *Phalocrama mitra* were discarded), but there was no strong imbalance toward one trophic type.

Model ensembles: Ensembles models were created by stacking all the individual models selected per month and trophic types and calculating an arithmetic mean between all the layers. No weighting was applied.

Analysis and Correction of non-independence

Spatial autocorrelation: Aggregation of occurrences was done on a 1° x 1° resolution.

Threshold selection

Threshold selection: Continuous maps were transformed into binary maps thanks to the threshold that was meeting the maximum Jaccard index for each species.

Assessment

Performance statistics

Metric choice:

The Jaccard index was chosen here to evaluate the performance of the individual species distribution models because it is not influenced by differences in species prevalence [10]. This index is also a good way to evaluate the overlap between observed occurrences and predicted occurrences by models, in a presence-absence context. Models' results could indeed be divided in two parts: real occurrences (True predicted presences are True Positive (TP) and unpredicted true presences are False Negatives (FN)) and predicted occurrences (True predicted presences (TP) and False predicted presences are False Positives (FP)). For each individual model, a threshold between 0 and 1000 was set and the Jaccard index was calculated as TP/(TP + FN + FP) for each threshold. The maximum index was finally retained, and the corresponding threshold was used to create binary maps out of continuous maps.

Performance on training data: Maximum Jaccard index with a cutoff set to 0.3

Performance on validation data: Maximum Jaccard index with a cutoff set to 0.3

Plausibility check

Response shapes: We visually inspected the individual response curves for each predictor per species.

Prediction

Prediction output

Prediction unit: Monthly habitat suitability per trophic types estimated through the mean of habitat suitability indices per species that display this trophic type.

Uncertainty quantification

Algorithmic uncertainty: We used 5 different types of modeling approaches (GLM, GAM, MARS and ANN) combined in ensemble models to account for model choice uncertainty in our predictions.

Input data uncertainty: For each species, data uncertainty was taken into account by running 5 cross-validation per algorithm and per month.

Global uncertainty: For each species, uncertainty maps were built by calculating the standard deviation among the suitability indices of each ensemble model member. This way we identified the regions where models results are consensual or divergent.

Novel environments: -

Discussion about SDMs reliability:

Our SDMs rely on four assumptions: (i) they assume that modelled environmental niches and associated traits are conserved over time ("niche conservatism"; [11], (ii) they assume unlimited dispersal by sea currents and (iii) they assume that the niches modelled are not constrained by biotic interactions and (iv) occurrence data (absence/presence) reflects the imprint of the abundance and then fitness. The first assumption is not likely to have a strong impact on the results presented above, as our study focuses on contemporary patterns and not future projections [12], built with annually averaged occurrence data and climatologies that aimed to purposely smooth out the species' population dynamics on finer scales. Moreover, the period covered by the observations (2002-2018) may be too short to integrate adaptation mechanisms that may have happened within the populations of the studied species. We argue that the data used to train the SDMs actually already integrate the outcome of biotic interactions that occurred in situ. Therefore, we cannot ignore that our SDMs indirectly integrate the imprint of such interactions in their response curves and therefore in their HSI projections We encourage future modelling studies about protists trophic traits biogeography to focus on species interactions, especially in the case of phagotrophy and predation, using Joint-Species Distribution Models [13]. Cell dispersal driven by water masses connectivity is another major process that shapes spatial distribution of marine protists [14–16]. In our case, protists are supposed to have globally large and abundant populations, they are then less impacted by dispersal by oceanic currents [17]. Moreover, open-ocean regions are strongly connected at large scale, tempering the dispersal effect on dinoflagellates biogeography [16]. Finally, the modelled distribution here is based on presence/absence of species, smoothing out the abundance of populations in the models, which could misrepresent the fitness of species or traits. However, the similar evenness profiles between the three trophic strategies reflects that there is no major misrepresentation of one trophic strategy when converting abundance to presence. Integrating abundance as the explained variable of the models would be however challenging due to the numerous gene copies in dinoflagellates, resulting in a biased correspondence between reads and cells absolute number [18]. The pipeline developed here is

however reproducible and as we worked at the species level, the outcome of modeling doesn't intrinsically depend on this trophic annotation, which could be modified according to future dedicated works.

Species	Environmental predictor	Trophic type	Mixotrophic type	Outputs used
Abedinium_dasypus	si MLD Sistar no3	phagotroph	-	*
Alexandrium_affine	SST chla po4 Sistar	mixotroph	CM	
Alexandrium_andersonii	SST chla si PAR	mixotroph	CM	*
Alexandrium_hiranoi	SST chla po4	phototroph	-	
Alexandrium_ostenfeldii	SST chla no3 MLD	mixotroph	CM	
Amphidinium_longum	PAR MLD SST no3 si po4 Sistar chla	phagotroph	-	*
Amphidoma_languida	SST PAR	phototroph	-	*
Ansanella_granifera	Sistar MLD PAR po4	mixotroph	CM	
Azadinium_caudatum	SST chla Sistar po4	phototroph	-	*
Azadinium_dexteroporum	chla Sistar SST	phototroph	-	*
Azadinium_obesum	SST si pic	phototroph	-	
Azadinium_poporum	SST no3 PAR	phototroph	-	*
Azadinium_trinitatum	SST PAR si Sistar pic chla	phototroph	-	*
Biecheleria_cincta	Sistar SST si PAR MLD no3	mixotroph	CM	*
Dinophysis_acuminata	SST si chla pic PAR Sistar no3	mixotroph	pSNCM	
Erythropsidinium_agile	SST MLD	phagotroph	-	*
Fragilidium_sp.	no3 SST po4	mixotroph	CM	*
Gonyaulax_polygramma	SST chla	mixotroph	CM	*
Gonyaulax_spinifera	SST chla	mixotroph	CM	
Gymnodinium_aureolum	MLD SST	mixotroph	CM	*
Gymnodinium_smaydae	SST Sistar MLD si	mixotroph	CM	*
Gyrodinium_dominans	SST	phagotroph	-	*
Gyrodinium_fusiforme	chla MLD no3	phagotroph	-	*
Gyrodinium_helveticum	MLD Sistar po4 SST	phagotroph	-	*
Gyrodinium_heterogrammum	SST no3 MLD si chla	phagotroph	-	*
Gyrodinium_rubrum	SST MLD	phagotroph	-	*
Gyrodinium_spirale	no3 po4	phagotroph	-	*
Heterocapsa_nei_rotundata	SST no3 MLD Sistar PAR	mixotroph	-	*
Heterocapsa_triquetra	MLD SST PAR	mixotroph	CM	*
Islandinium_tricingulatum	no3 po4 Sistar PAR chla SST	phagotroph	-	*
Karenia_bicuneiformis	SST chla	mixotroph	CM	
Karenia_brevis	SST chla MLD po4 Sistar pic	mixotroph	CM	*
Karenia_mikimotoi	SST Sistar po4 PAR chla MLD	mixotroph	CM	
Karlodinium_sp.	SST	mixotroph	CM	*
Karlodinium_veneficum	SST si PAR MLD Sistar no3	mixotroph	CM	*
Lepidodinium_chlorophorum	SST Sistar	mixotroph	CM	*
Lepidodinium_sp.	SST Sistar chla no3	mixotroph	CM	*
Lessardia_elongata	SST no3 pic MLD po4	phototroph	-	*
Levanderina_fissa	SST Sistar no3	mixotroph	CM	
Margalefidinium_fulvescens	SST MLD	mixotroph	CM	*
Margalefidinium_polykrikoides	SST chla po4 si	mixotroph	CM	*
Ornithocercus_quadratus	SST	mixotroph	eSNCM	*
Paragymnodinium_shiwhaense	po4 SST MLD no3 PAR Sistar chla	mixotroph	CM	*
Pentapharsodinium_sp.	si no3 Sistar chla	phototroph	-	*
Pentapharsodinium_tyrrhenicum	SST po4	mixotroph	CM	*
Phalacroma_mitra	SST chla	mixotroph	pSNCM	
Polykrikos_kofoidii	SST PAR	phagotroph	-	*
Prorocentrum_cordatum	PAR po4 Sistar chla MLD no3 pic	mixotroph	CM	*
Prorocentrum_donghaiense	SST PAR	mixotroph	CM	*
Prorocentrum_triestinum	si po4	mixotroph	CM	
Proterythropsis_sp.	SST Sistar MLD si	phagotroph	-	*
Protoceratium_reticulatum	po4 SST MLD	mixotroph	CM	*
Protoperidinium_bipes	SST no3 po4 chla pic Sistar	phagotroph	-	*
Protoperidinium_depressum	SST chla	phagotroph	-	*
Protoperidinium_elegans	po4 SST Sistar	phagotroph	-	*
Protoperidinium_pellucidum	Sistar SST MLD	phagotroph	-	
Protoperidinium_punctulatum	SST PAR	phagotroph	-	*
Protoperidinium_sp.	po4 si MLD SST chla Sistar PAR	phagotroph	-	*
Pyrocystis_lunula	SST po4 PAR chla	phototroph	-	*
Pyrocystis_sp.	SST po4	phototroph	-	*
Scrippsiella_acuminata	SST Sistar si PAR	mixotroph	CM	*
Sinophysis_stenosoma	SST MLD Sistar	phagotroph	-	*
Spiniferodinium_palauense	SST	mixotroph	CM	*
Spiniferodinium_palustre	SST	mixotroph	CM	*
Symbiodinium_sp.	SST chla	mixotroph	CM	*
Takayama_pulchellum	SST Sistar MLD	mixotroph	CM	*
Torodinium_robustum	SST	mixotroph	CM	*
Torodinium_teredo	PAR chla	mixotroph	CM	*
Tripos_furca	SST Sistar MLD PAR po4 chla si	mixotroph	CM	*
Tripos_fusus	SST chla Sistar PAR	mixotroph	CM	*
Warnowia_sp.	SST si	phagotroph	-	*
Yihiella_yeosuensis	SST si PAR	mixotroph	CM	

Table S8: List of species with the corresponding environmental predictors, used for calibration of individual models. For each species, the column "Output used" indicates if the species individual models' were used for building final trophic maps. (* if yes, otherwise no).

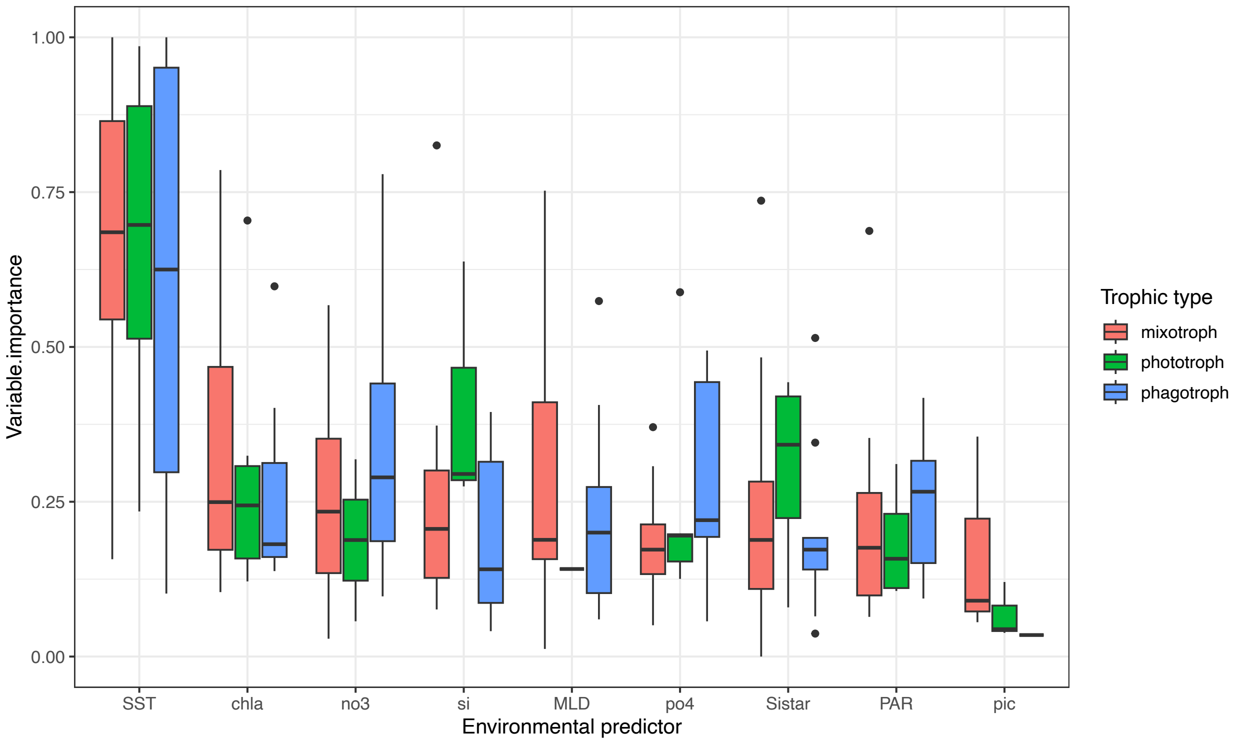

Figure S9: Distribution of the scores of relative variable importance computed for each dinoflagellates species during model calibration and summarized for the three trophic types. SST: Sea Surface Temperature (°C), chla: Surface Chlorophyll-a concentration (mg.m-3), no3: surface nitrates Concentration (µmol.kg-1), si: Surface Silicates Concentration (µmol.kg-1), MLD: Mixed Layer Depth (m), po4: Surface Phosphates Concentration (µmol.kg-1), Sistar: Excess of Silicates relative to Nitrates, PAR: Photosynthetically Active Radiation (µmol.m-2.s-1), pic: Surface Particulate Inorganic Carbon Concentration (mol.m-3).

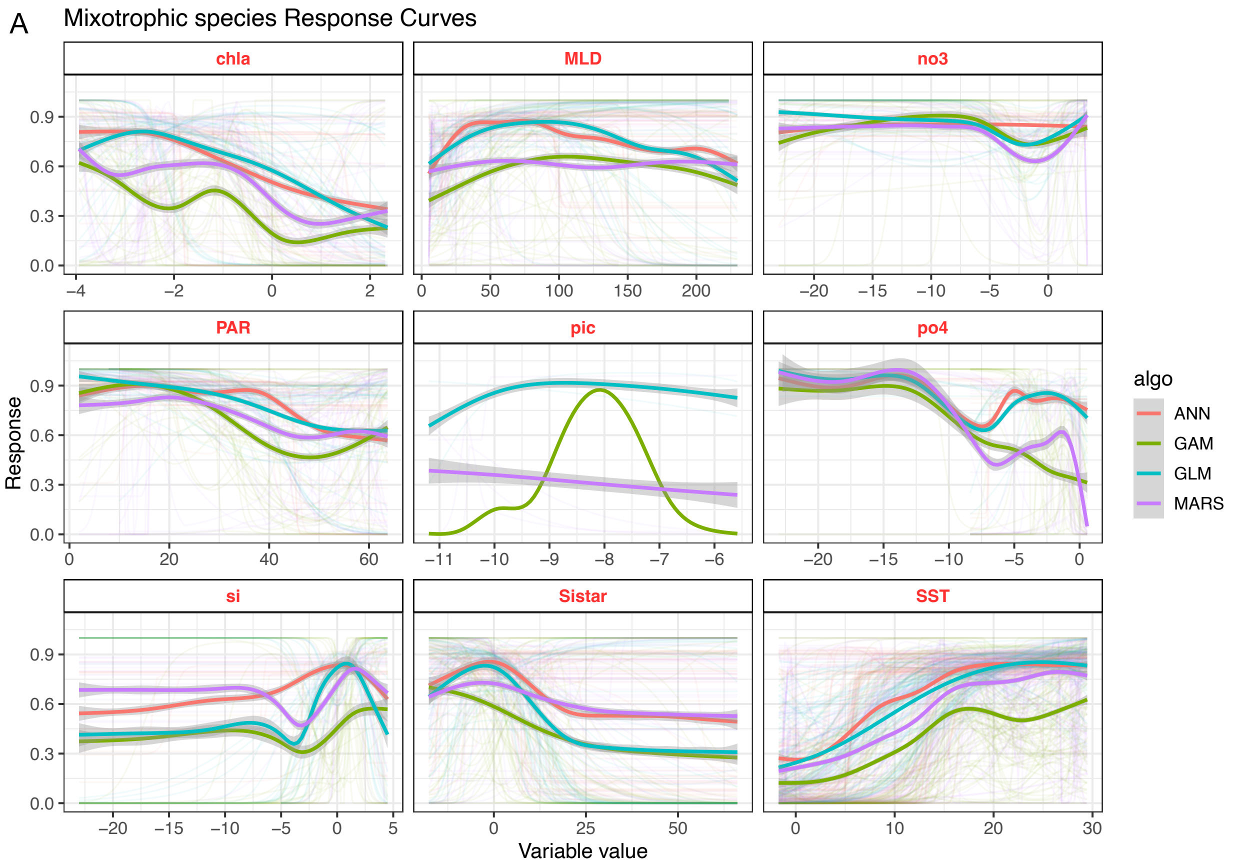

Fig. S10: A: Response curves for the 9 environmental predictors used to calibrate the individual models corresponding to mixotrophic species.

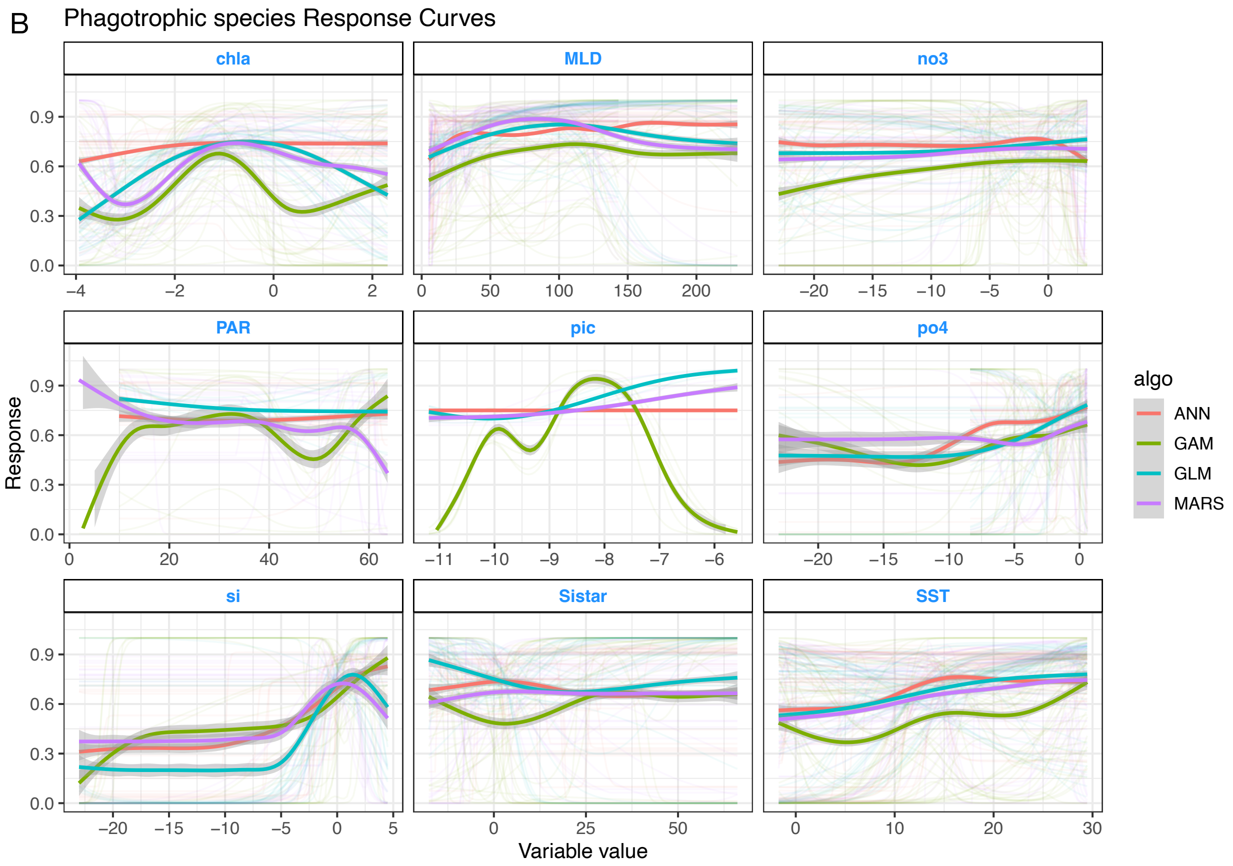

B: Response Curves for the 9 environmental predictors used to calibrate the individual models corresponding to phagotrophic species.

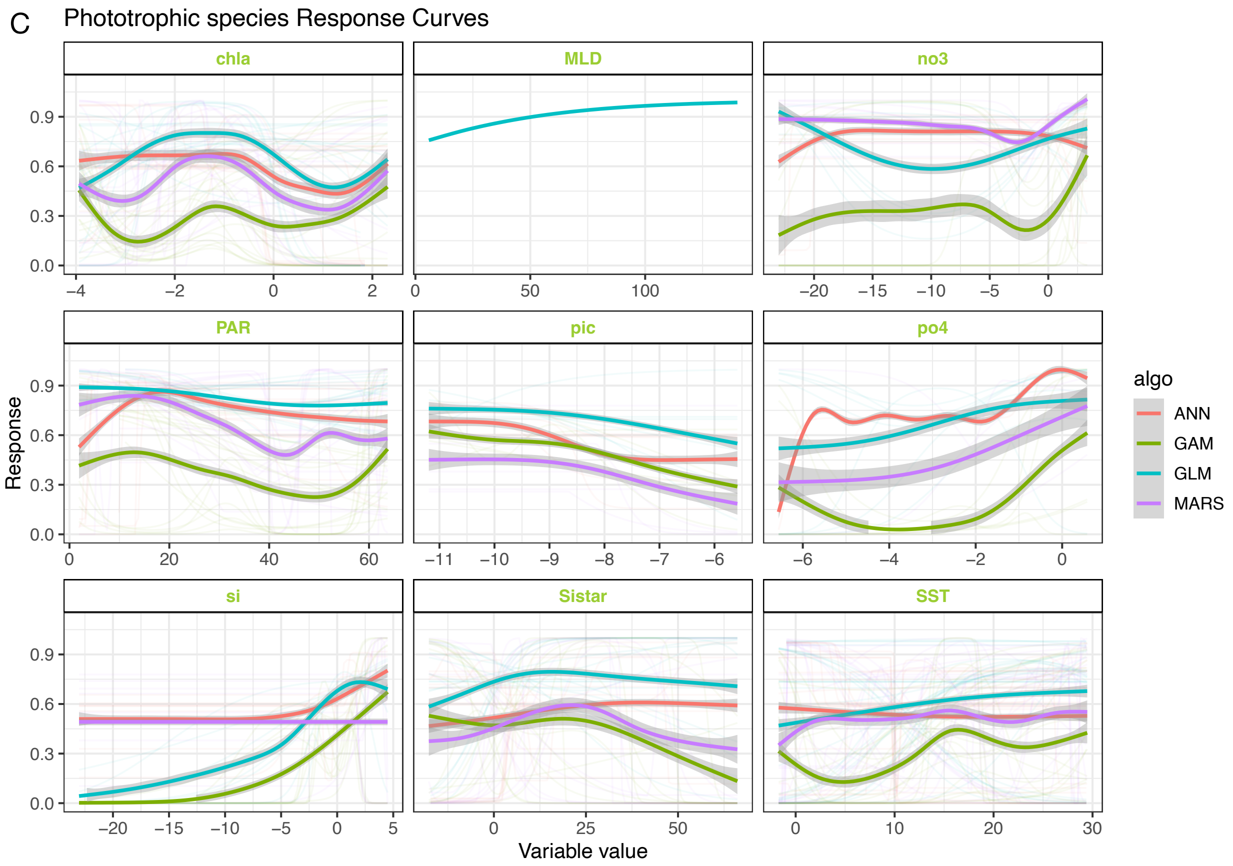

C: Response Curves for the 9 environmental predictors used to calibrate the individual models corresponding to phototrophic species.

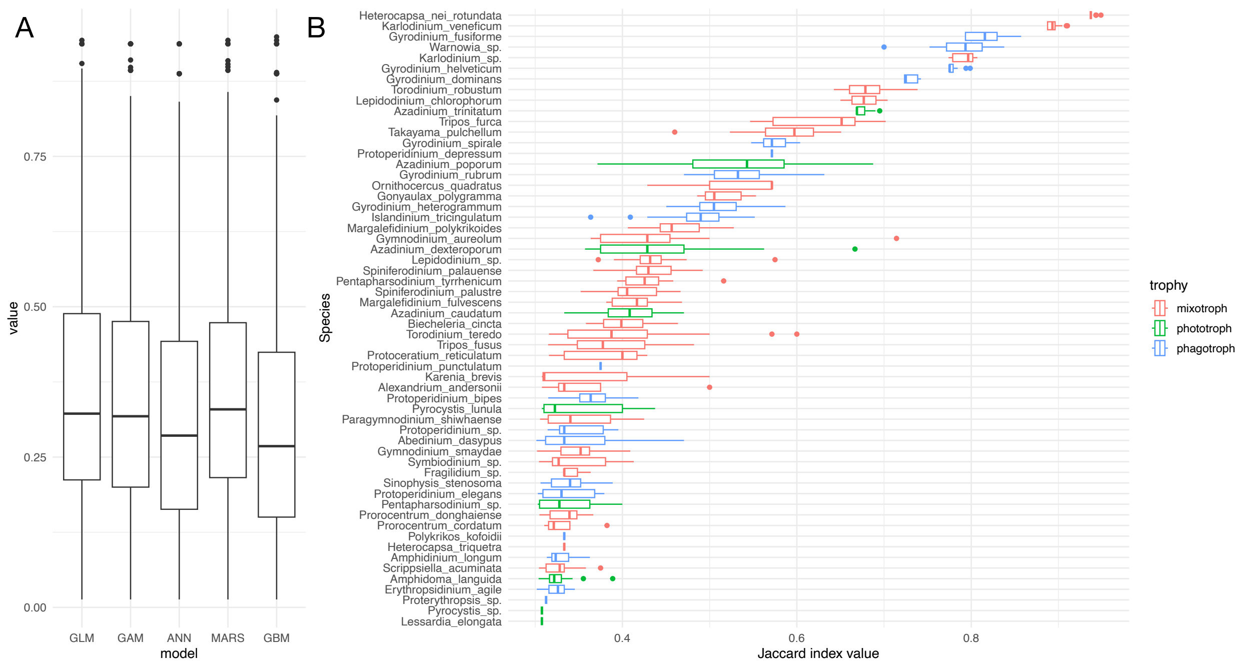

Figure S11: Jaccard scores for individual models. Figure A represents the relative performance of every modeling technique in terms of Jaccard indices. Figure B represents the distribution of Jaccard indices for the retained individual models after threshold selection (Jaccard > 0.3), depending on the trophic type of the species. (In total, 879 individual models were retained, 372 for mixotrophic species, 289 for strict phagotrophic species and 218 for strict phototrophic species, corresponding to 58 out of 72 species modeled)

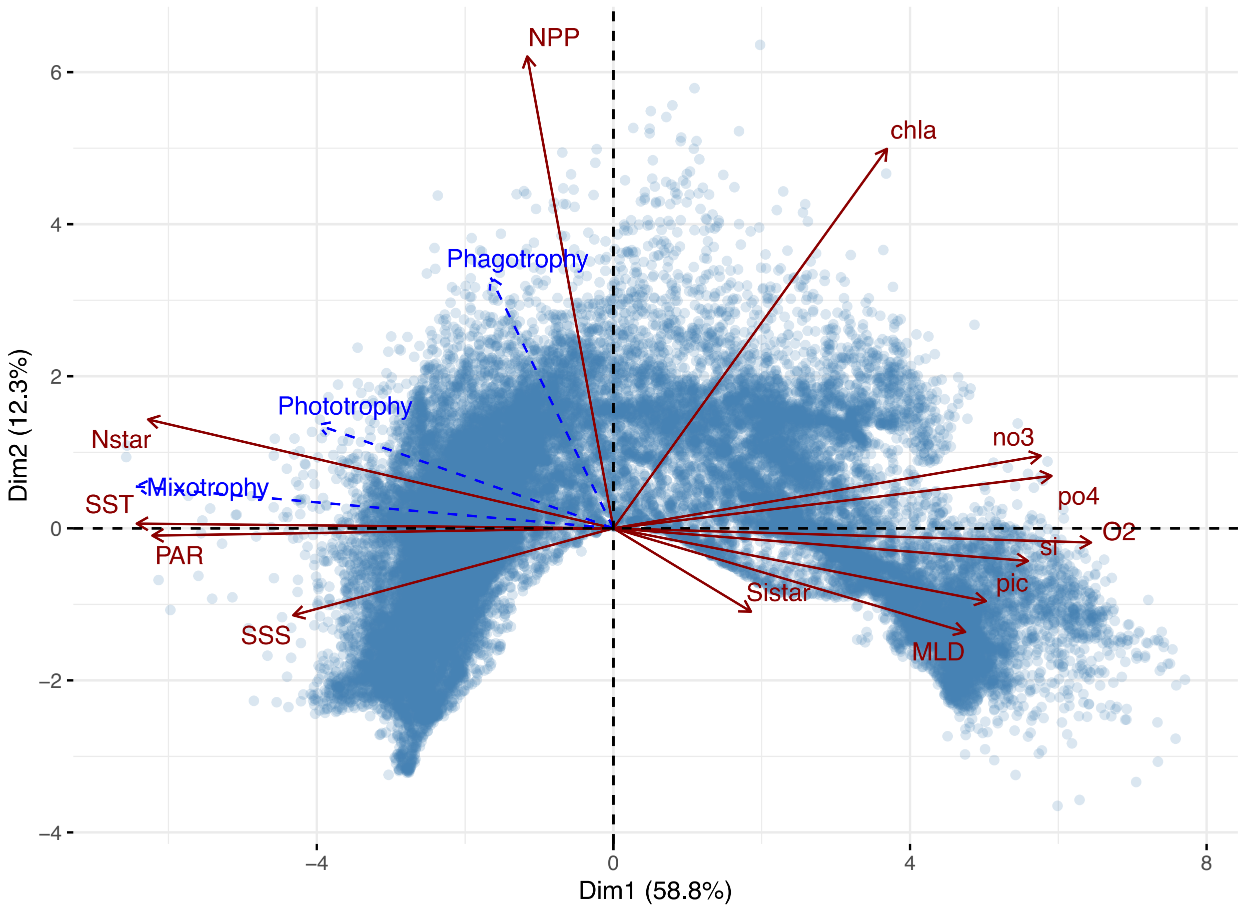

Figure S12: Principal component analysis computed on mean habitat suitability (HSI) values of each mixotrophic species, with environmental supplementary variables. Individual scores are projected onto the principal components, where each point represents a 1°×1° grid cell in the open ocean. Each individual represents a 1°x1° grid cell of the open-ocean. This figure complements Figures 4B, 5A–C, and S14.

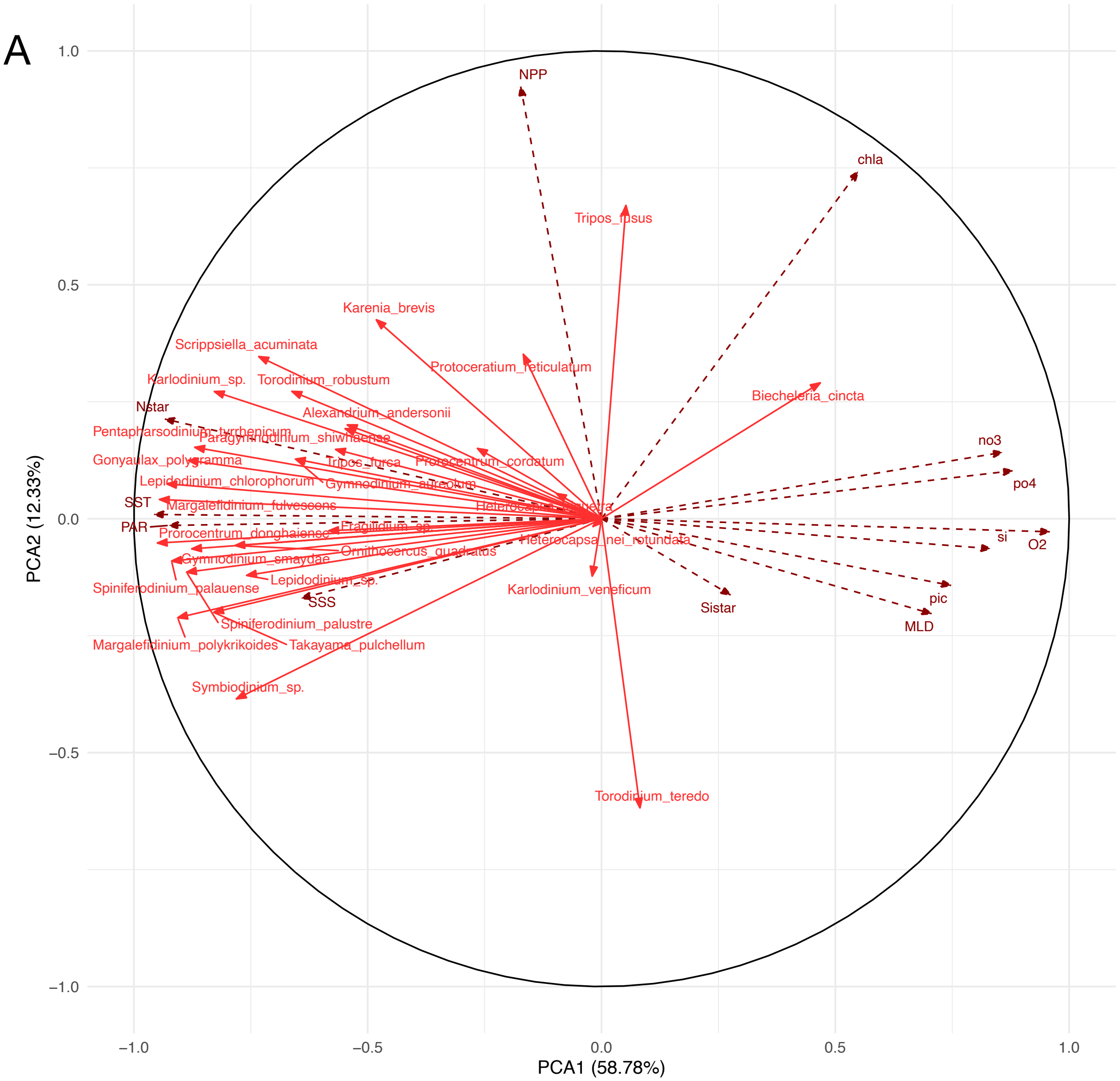

Figure S13: Principal component analysis computed on mean habitat suitability (HSI) values of each mixotrophic species, with environmental supplementary variables.

A: Mixotrophic species. The HSI is displayed within the open-ocean environmental space. Dark red arrows correspond to environmental predictors. Red arrows correspond to the species, plotted as supplementary variables. (n.b.: there is one eSNCM species, *Ornithocercus quadratus*).

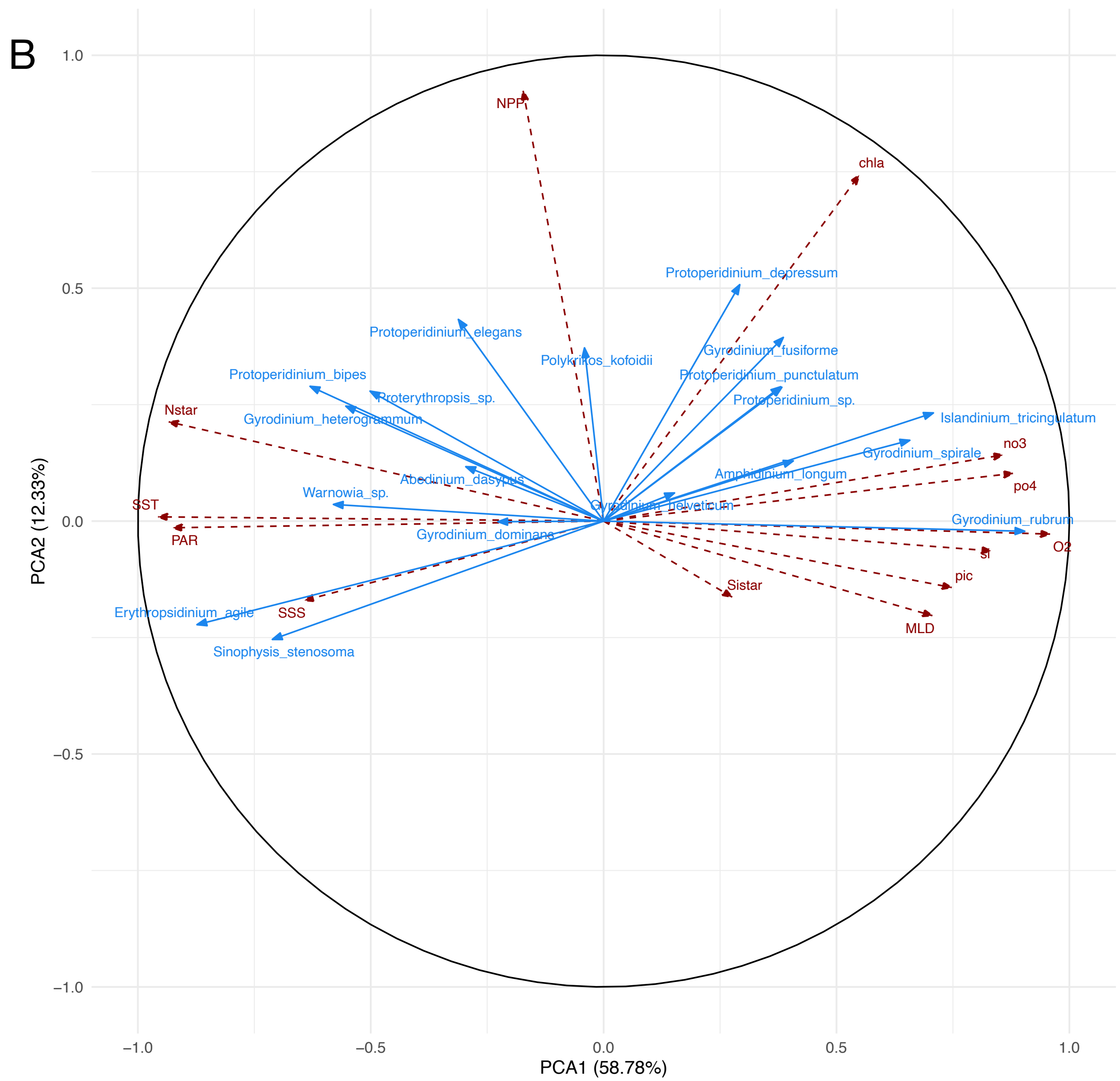

B: Strict phagotrophic species. The HSI is displayed within the open-ocean environmental space. Dark red arrows correspond to environmental predictors. Blue arrows correspond to the species, plotted as supplementary variables.

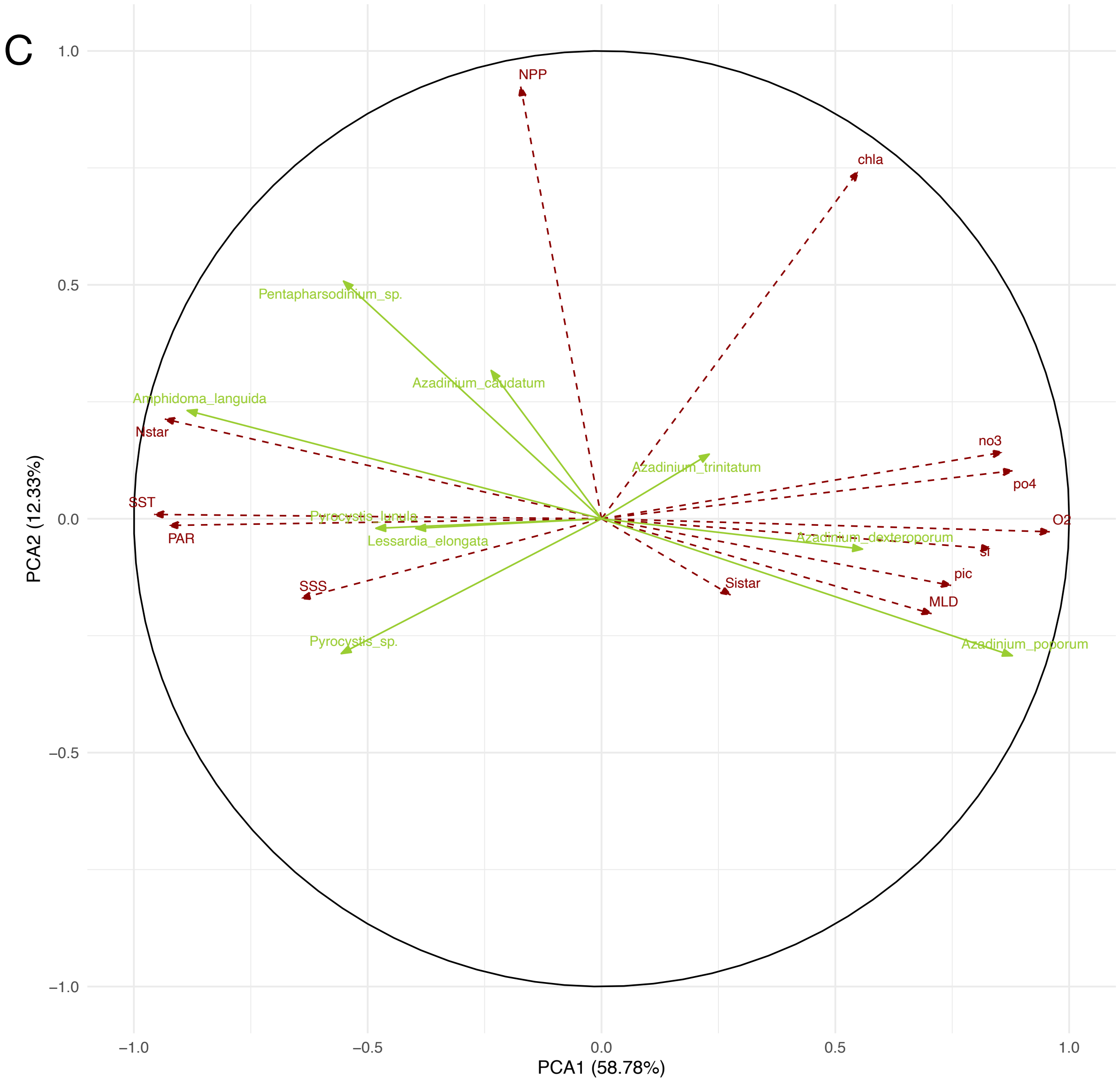

C: Strict phototrophic species. The HSI is displayed within the open-ocean environmental space. Dark red arrows correspond to environmental predictors. Green arrows correspond to the species, plotted as supplementary variables.

prov	group1	group2	n1	n2	statistic	p	p.adj	p.adj.signif
HIL	mixotroph	phototroph	14,337	14,337	88,912,844	0	0	****
HIL	mixotroph	phagotroph	14,337	14,337	45,189,018.000	0	0	****
HIL	phototroph	phagotroph	14,337	14,337	55,409,448.000	0	0	****
HIT	mixotroph	phototroph	3,860	3,860	8,830,482	0	0	****
HIT	mixotroph	phagotroph	3,860	3,860	9,407,160.000	0	0	****
HIT	phototroph	phagotroph	3,860	3,860	8,376,370	0	0	****
MTR	mixotroph	phototroph	2,631	2,631	6,168,828.000	0	0	****
MTR	mixotroph	phagotroph	2,631	2,631	5,525,906.000	0	0	****
MTR	phototroph	phagotroph	2,631	2,631	3,492,340.000	0.570	1	ns
PEU	mixotroph	phototroph	1,243	1,243	1,244,093	0	0	****
PEU	mixotroph	phagotroph	1,243	1,243	922,983.500	0	0	****
PEU	phototroph	phagotroph	1,243	1,243	381,484	0	0	****
SUS	mixotroph	phototroph	2,251	2,251	4,049,814	0	0	****
SUS	mixotroph	phagotroph	2,251	2,251	4,657,618	0	0	****
SUS	phototroph	phagotroph	2,251	2,251	3,557,712	0	0	****
TRP	mixotroph	phototroph	8,517	8,517	67,280,688.000	0	0	****
TRP	mixotroph	phagotroph	8,517	8,517	72,067,634.000	0	0	****
TRP	phototroph	phagotroph	8,517	8,517	57,607,876.000	0	0	****
WIS	mixotroph	phototroph	2,644	2,644	5,021,224.000	0	0	****
WIS	mixotroph	phagotroph	2,644	2,644	6,915,971	0	0	****
WIS	phototroph	phagotroph	2,644	2,644	6,170,362.000	0	0	****

Table S14: Pairwise Wilcoxon tests results of the HSI distribution comparison between trophic types across open-ocean biogeochemical provinces from [19].

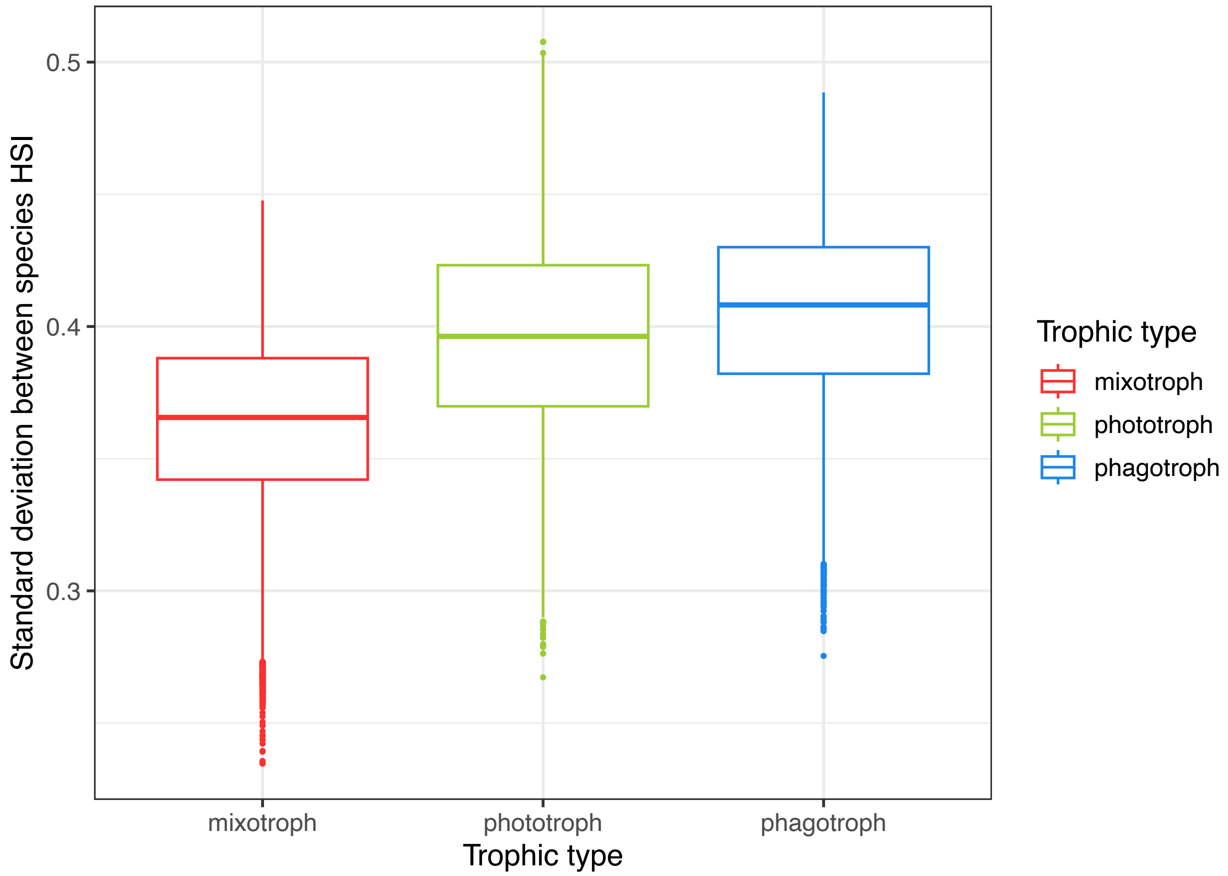

Figure S15: Boxplot of standard deviation between HSI of every species representing averaged trophic strategy at the open-ocean scale.

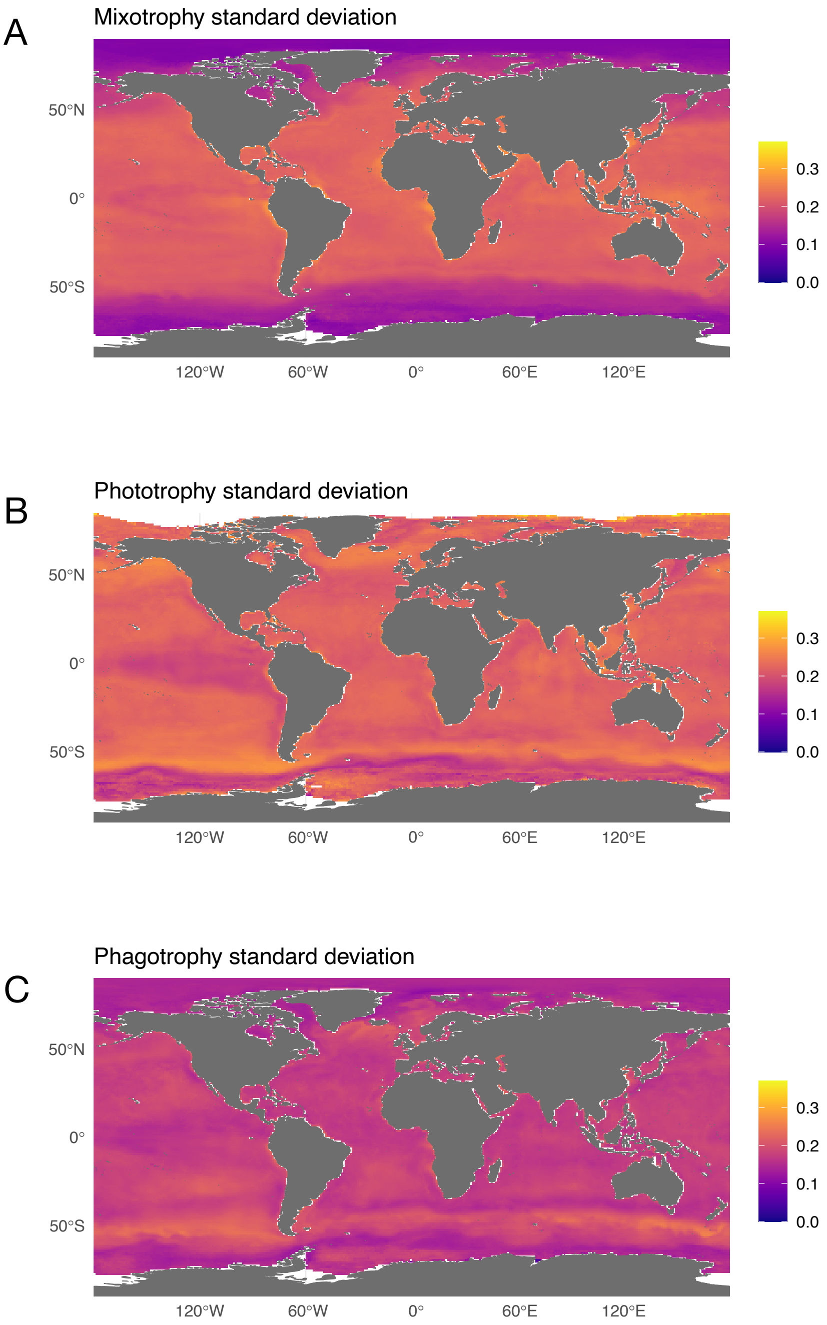

Figure S16: Maps of the standard deviation of individual models results computed for each ensemble models, averaged per monthly projection and per trophic types. A: Standard deviation of ensemble models results for mixotrophic species B: Standard deviation of ensemble models results for phototrophic species. C: Standard deviation of ensemble models results for strict phagotrophic species

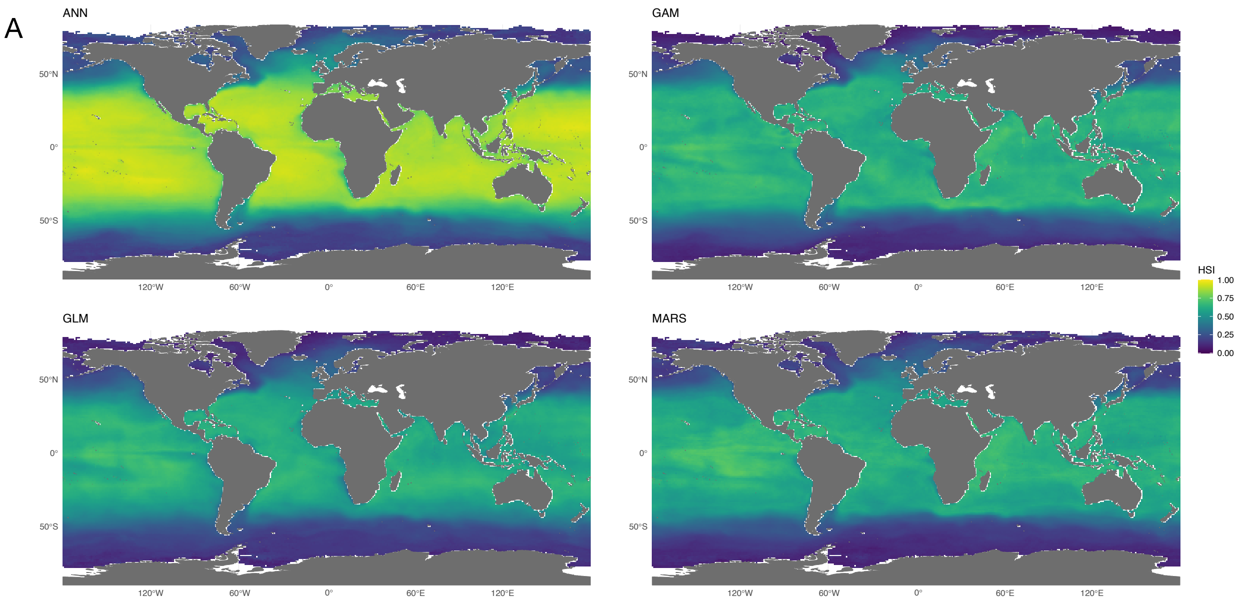
A
ANN
GAM
GLM
MARS
50°N
0°
50°S
120°W
60°W
0°
60°E
120°E
HSI
1.00
0.75
0.50
0.25
0.00

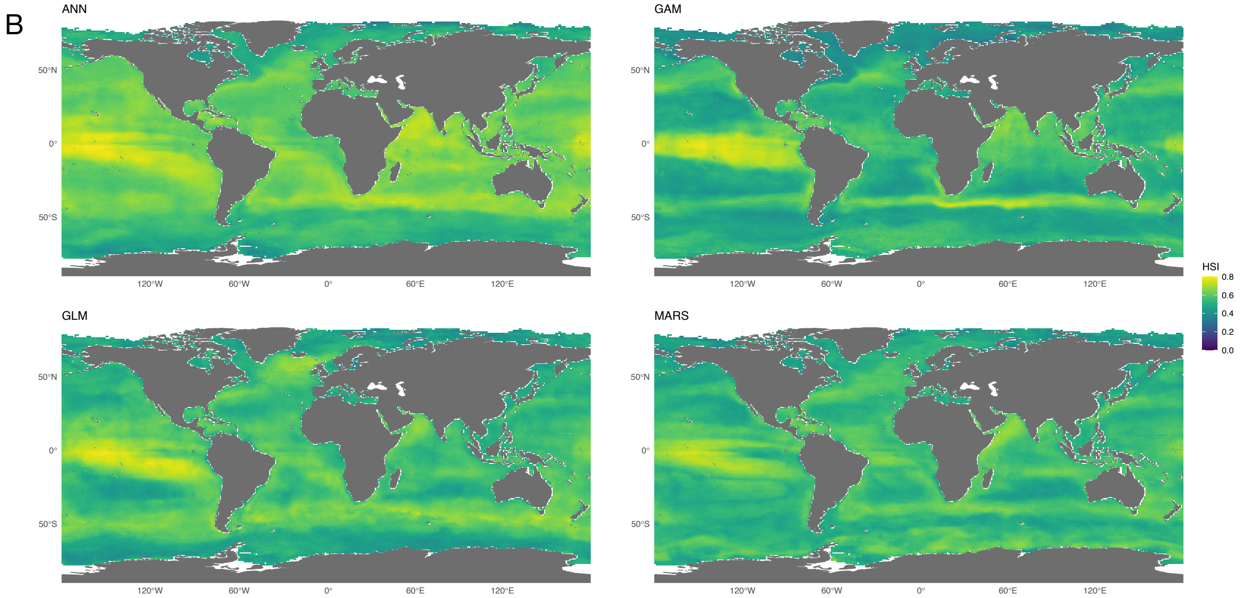
B
ANN
GAM
GLM
MARS
50°N
0°
50°S
120°W
60°W
0°
60°E
120°E
HSI
0.8
0.6
0.4
0.2
0.0

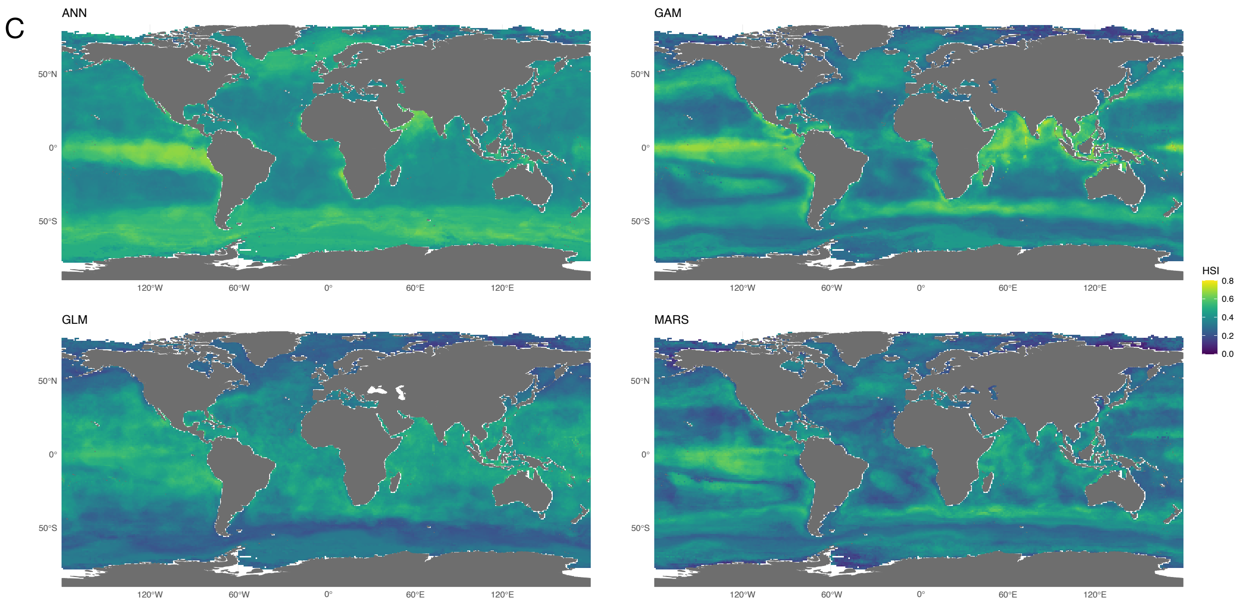

Figure S17: Maps of annually averaged habitat suitability index of A: mixotrophy, B: strict phagotrophy, C: strict phototrophy, depending on the modeling technique (ANN, GAM, GLM, MARS)

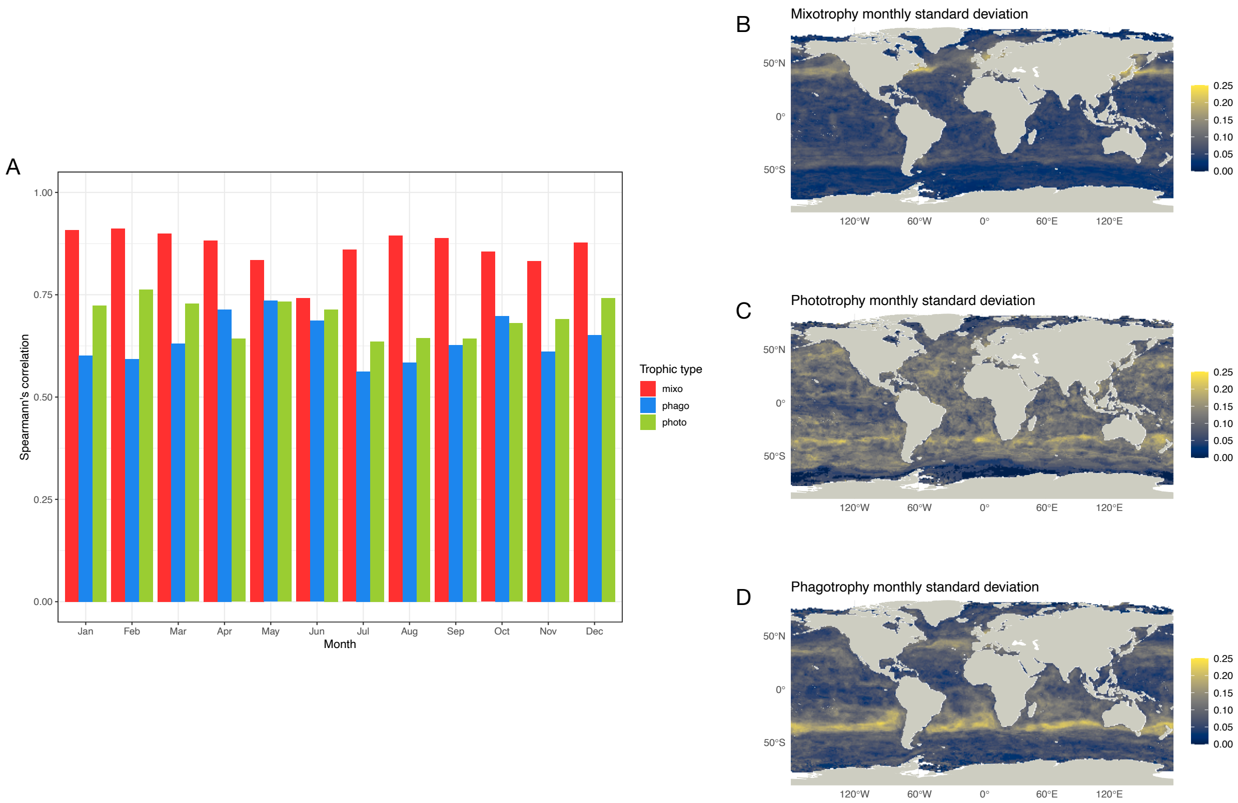

Figure S18: Monthly variability of habitat suitability index global distribution for the three trophic types.

A : Spearman's correlations between monthly averaged HSI maps and annual averaged HSI map for each monthly projection for the three trophic types

B : Standard-deviation of monthly averaged HSI distribution maps for mixotrophy.

C: Standard-deviation of monthly averaged HSI distribution maps for strict phototrophy.

D: Standard-deviation of monthly averaged HSI distribution maps for strict phagotrophy.

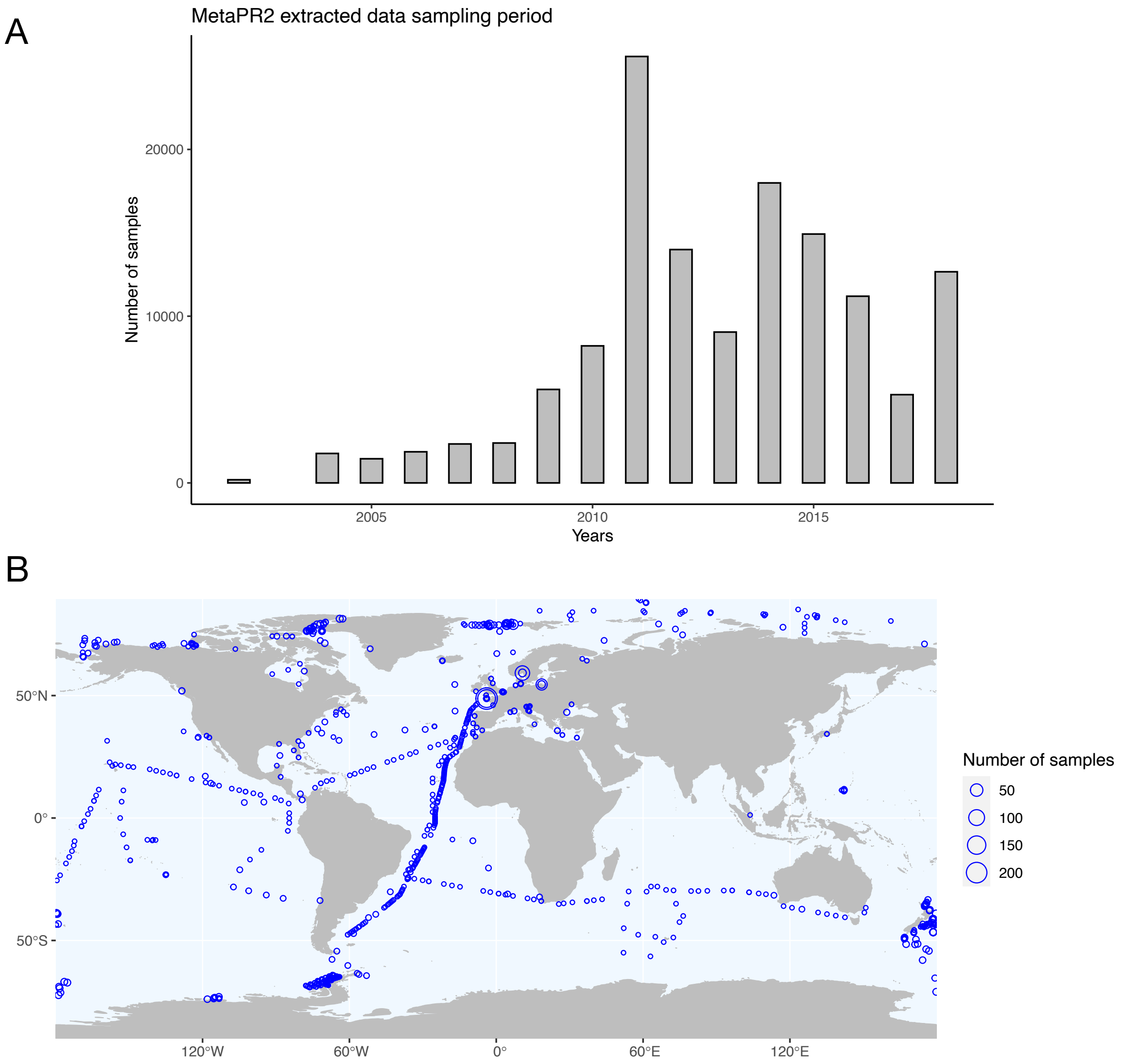

Figure S19: Temporal and spatial sampling effort for Dinophyceae ASVs from metaPR2-extracted dataset. **A:** Temporal evolution of the sampling effort for the Dinophyceae samples used in this study. **B:** Map of the Dinophyceae sampling locations used in this study. The size of the circle represents the number of samples.

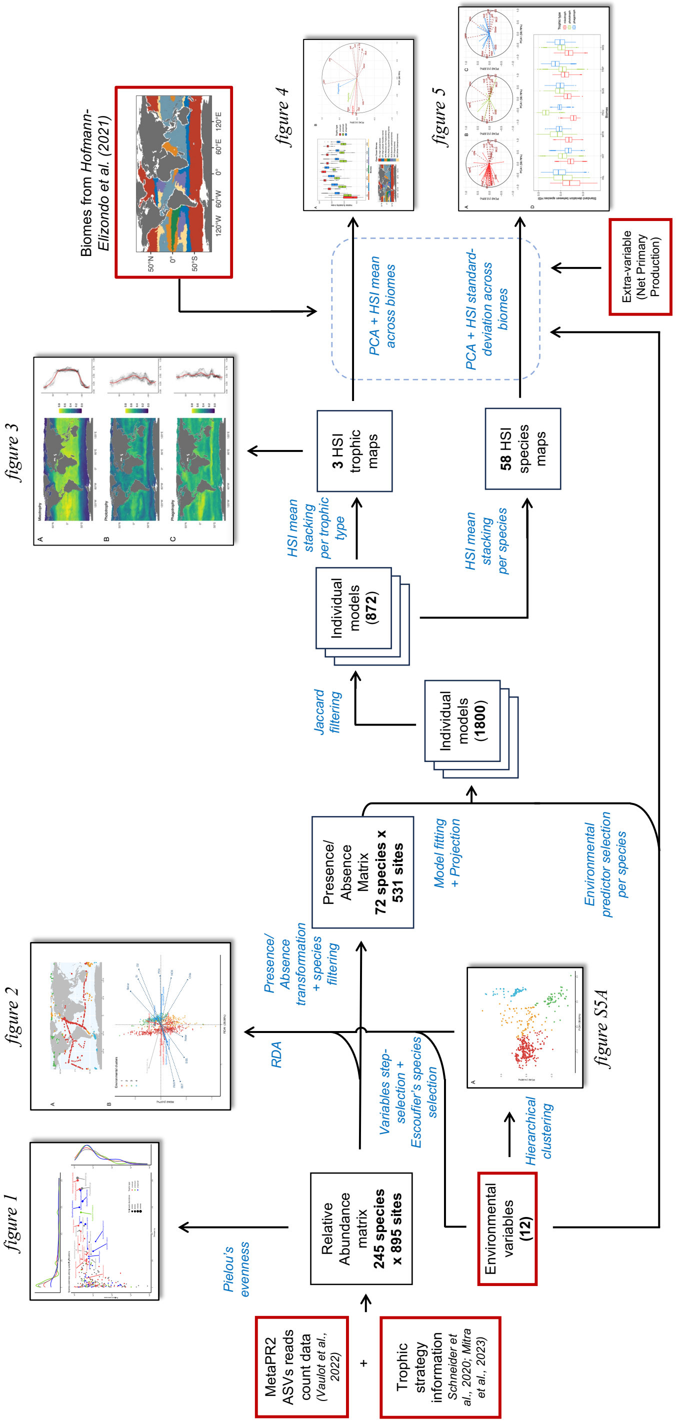

Figure S20: Schematic diagram of the workflow carried out in this study. Red squares represent the input data, and blue italic text indicates the analyses performed. References:

Schneider LK, Anestis K, Mansour J et al. A dataset on trophic modes of aquatic protists. Biodivers Data J 2020;8:e56648.
Mitra A, Caron DA, Faure E et al. The Mixoplankton Database (MDB): Diversity of photo-phago-trophic plankton in form, function, and distribution across the global ocean. Journal of Eukaryotic Microbiology 2023;70:e12972.
Hofmann Elizondo U, Righetti D, Benedetti F et al. Biome partitioning of the global ocean based on phytoplankton biogeography. Progress in Oceanography 2021;194:102530.

References

1. Zurell D, Franklin J, König C *et al.* A standard protocol for reporting species distribution models. *Ecography* 2020;**43**:1261–77.
2. Vaulot D, Sim CWH, Ong D *et al.* metaPR2: a database of eukaryotic 18S rRNA metabarcodes with an emphasis on protists. 2022:2022.02.04.479133.
3. R Core Team. R: A Language and Environment for Statistical Computing. 2019.
4. Hijmans RJ. terra: Spatial Data Analysis. 2020:1.8-21.
5. Hijmans RJ. raster: Geographic Data Analysis and Modeling. R package version 3.0-12. 2020.
6. Thuiller W, Georges D, Engler R *et al.* biomod2: Ensemble Platform for Species Distribution Modeling. R package version 3.4.6. 2020.
7. Pebesma E. Simple Features for R: Standardized Support for Spatial Vector Data. *The R Journal* 2018;**10**:439–46.
8. Knecht NS, Benedetti F, Hofmann Elizondo U *et al.* The Impact of Zooplankton Calcifiers on the Marine Carbon Cycle. *Global Biogeochemical Cycles* 2023;**37**:e2022GB007685.
9. Leroy B, Meynard CN, Bellard C *et al.* virtualspecies, an R package to generate virtual species distributions. *Ecography* 2016;**39**:599–607.
10. Leroy B, Delsol R, Hugueny B *et al.* Without quality presence–absence data, discrimination metrics such as TSS can be misleading measures of model performance. *Journal of Biogeography* 2018;**45**:1994–2002.
11. Wiens JJ, Ackerly DD, Allen AP *et al.* Niche conservatism as an emerging principle in ecology and conservation biology. *Ecology Letters* 2010;**13**:1310–24.
12. Benedetti F, Vogt M, Elizondo UH *et al.* Major restructuring of marine plankton assemblages under global warming. *Nat Commun* 2021;**12**:5226.
13. Pollock LJ, Tingley R, Morris WK *et al.* Understanding co-occurrence by modelling species simultaneously with a Joint Species Distribution Model (JSDM). *Methods in Ecology and Evolution* 2014;**5**:397–406.
14. Bendtsen J, Sørensen LL, Daugbjerg N *et al.* Phytoplankton diversity explained by connectivity across a mesoscale frontal system in the open ocean. *Sci Rep* 2023;**13**:12117.
15. Da Silva O, Ayata S-D, Ser-Giacomi E *et al.* Genomic differentiation of three pico-phytoplankton species in the Mediterranean Sea. *Environmental Microbiology* 2022;**24**:6086–99.
16. Jönsson BF, Watson JR. The timescales of global surface-ocean connectivity. *Nat Commun* 2016;**7**:11239.
17. Villarino E, Watson JR, Jönsson B *et al.* Large-scale ocean connectivity and planktonic body size. *Nat Commun* 2018;**9**:142.
18. Biard T, Bigeard E, Audic S *et al.* Biogeography and diversity of Collodaria (Radiolaria) in the global ocean. *The ISME Journal* 2017;**11**:1331–44.
19. Hofmann Elizondo U, Righetti D, Benedetti F *et al.* Biome partitioning of the global ocean based on phytoplankton biogeography. *Progress in Oceanography* 2021;**194**:102530.
